# Chromosome-level genome assembly of *Origanum vulgare* subsp. *hirtum* reveals evolutionary insights and regulatory modules in terpenoid biosynthesis

**DOI:** 10.1093/hr/uhag030

**Published:** 2026-01-30

**Authors:** Tingchao Yin, Hefeng Guo, Yaolong Zhu, Yicheng Yang, Huanhuan Hao, Xinbao Liu, Junhao Lou, Caiyi Xie, Ya Wang, Haidong Yan, Linkai Huang, Yuzhu Li, Shuo Yan, Yingjun Chi, Bin Xu, Jing Zhang

**Affiliations:** College of Agro-grassland Science, Nanjing Agricultural University, Nanjing 210095, China; College of Agro-grassland Science, Nanjing Agricultural University, Nanjing 210095, China; College of Agro-grassland Science, Nanjing Agricultural University, Nanjing 210095, China; College of Agro-grassland Science, Nanjing Agricultural University, Nanjing 210095, China; College of Agro-grassland Science, Nanjing Agricultural University, Nanjing 210095, China; College of Agro-grassland Science, Nanjing Agricultural University, Nanjing 210095, China; College of Agro-grassland Science, Nanjing Agricultural University, Nanjing 210095, China; College of Agro-grassland Science, Nanjing Agricultural University, Nanjing 210095, China; Kunming Institute of Botany, Chinese Academy of Sciences, Kunming 650201, China; College of Grassland Science and Technology, Sichuan Agricultural University, Chengdu 611130, China; College of Grassland Science and Technology, Sichuan Agricultural University, Chengdu 611130, China; Key Laboratory of Grassland Ecosystem of the Ministry of Education, Gansu Agricultural University, Lanzhou 730070, China; Zhiguang Cryptic (Jiangsu) Biotechnology Co., Ltd., Nantong 226200, China; College of Agro-grassland Science, Nanjing Agricultural University, Nanjing 210095, China; College of Agro-grassland Science, Nanjing Agricultural University, Nanjing 210095, China; Key Laboratory of Grassland Ecosystem of the Ministry of Education, Gansu Agricultural University, Lanzhou 730070, China; College of Agro-grassland Science, Nanjing Agricultural University, Nanjing 210095, China

## Abstract

Oregano (*Origanum vulgare*) is a highly valued aromatic herb for culinary, medicinal, and ornamental purposes. Its commercial value is largely from its essential oil (EO), which is rich in key bioactive terpenoids, such as carvacrol and thymol. Greek oregano (*O. vulgare* subsp. *hirtum*) subspecies is particularly prized for its high EO content. In this study, we generated a high-quality genome assembly of Greek oregano to investigate its evolutionary trajectory and the genetic basis of terpenoid biosynthesis. The assembly spans 709.74 Mb and is anchored to 15 chromosomes, with a scaffold N50 of 46.36 Mb. Comparative genomic analysis revealed a whole-genome duplication event, estimated at ~59.93 million years ago, which likely contributed to the diversification of terpenoid biosynthesis pathways within the Lamiaceae family. Using a rapid screening approach, we identified Greek oregano mutants with higher EO content. Integrated genomic and transcriptomic analysis of a high-EO mutant highlighted the importance of α-linolenic acid metabolism/jasmonic acid (JA) biosynthesis pathways in EO production. Exogenous JA treatment led to upregulation of key EO biosynthetic genes and higher EO content. Furthermore, a JA-inducible bHLH transcription factor, OvbHLH13, was identified as a central regulator of terpenoid biosynthesis. Through Y1H, transcriptional activation, and EMSA assays, we demonstrated that OvbHLH13 directly bound to and transactivated the promoter of *OvSDR1*, which encodes a critical enzyme in thymol and carvacrol production. Collectively, this genomic resource provides valuable insights into the genetic and regulatory network controlling terpenoid biosynthesis and establishes a critical genomic foundation for molecular breeding of Greek oregano.

## Introduction

Oregano (*O. vulgare*), a member of the Lamiaceae family native to Eurasia and North Africa, is a globally popular aromatic herbs, valued for culinary, herbal medicine, and ornamental purposes [1–2]. Oregano’s commercial importance is largely attributed to its essential oils (EOs) complex composed predominantly of monoterpenes (e.g., carvacrol and thymol) and phenolic compounds that confer strong antimicrobial, antioxidant, and flavor-enhancing properties [3–5]. With growing global concerns over antibiotic overuse in livestock production (e.g., China’s ‘antibiotic-free feedstock’ policy effective since 2020), oregano EO has emerged as a safe and effective alternative, owing to its ability to inhibit a broad spectrum of pathogenic bacteria such as *Staphylococcus aureus*, *Streptococcus*, *Escherichia coli*, and *Salmonella* [1–3]. The *O*. *vulgare* comprises six recognized subspecies: *O*. *vulgare* subsp. *vulgare*, subsp. *gracile*, subsp. *virens*, subsp. *hirtum*, subsp. *viridulum*, and subsp. *glandulosum* [6]. Notably, Greek oregano (*O*. *vulgare* subsp. *hirtum*) exhibits higher EO content than other subspecies [6]. Varieties/accessions of Greek oregano with higher carvacrol and thymol are considered the better quality [7, 8]; however, the key genetic basis and regulatory mechanisms of terpenoid biosynthesis in this economically important subspecies remain largely uncharacterized.

Current understanding of the biosynthesis of terpenoids (e.g., carvacrol and thymol) involves the mevalonate (MVA) and methylerythritol phosphate (MEP) pathways [4]. Briefly, these pathways first generate isopentenyl diphosphate (IPP) and dimethylallyl diphosphate (DMAPP), which are then converted by short-chain prenyltransferases into geranyl diphosphate (GPP) and farnesyl diphosphate (FPP). GPPs are then cyclized by terpene synthase (TPSs) to form γ-terpinene, which is subsequently oxidized by multiple CYP71D subfamily P450 monooxygenases to produce unstable cyclohexadienol intermediates. Finally, these intermediates undergo dehydrogenation catalyzed by short-chain dehydrogenase/reductase (SDR) to form ketones (e.g., ketone carvone), followed by keto-enol tautomerism to yield thymol and carvacrol. Genes coding for key enzymes in this pathway have been characterized in thyme (*Thymus vulgaris*) and oregano, including *TvSDR1*, *TvCYP71D179*, *TvCYP71D180*, *TvCYP71D507*, and *TvTPS2* in thyme and *OvCYP71D178* and *OvCYP71D181* in oregano [2, 4]. For example, three thyme CYP71D monooxygenases (TvCYP71D179, TvCYP71D180, and TvCYP71D507) oxidize γ-terpinene to generate unstable cyclohexadienol intermediates, which are subsequently dehydrogenated by TvSDR1 to yield thymol and carvacrol [4]. In oregano, OvCYP71D178 converts γ-terpinene to thymol, whereas OvCYP71D181 and TvCYP71D182 are involved in carvacrol production [4]. Nevertheless, additionally key enzymes (e.g., SDR, TPS, and CYP450 family members) involved in the biosynthesis of thymol, carvacrol, and other terpenoids in oregano remain to be cloned and functionally characterized.

Beyond the core enzymatic machinery, the expression of terpenoid biosynthesis genes in oregano is subject to tight regulatory control [2, 4]. However, the complete set of genes involved in this pathway and their underlying transcriptional regulatory mechanisms remain largely unclear. In contrast, studies on other medicinal and aromatic plant species have identified several transcription factors (TFs) as key regulators of terpenoid biosynthesis by regulating genes in MVA and MEP pathways [9–14]. For instance, in *Artemisia annua*, co-activation of *amorpha-4,11-diene synthase* gene by both AabHLH1 and AabZIP1 enhances artemisinin biosynthesis [9]. In *Santalum album*, the AREB family TF SaAREB6 specifically binds to the AREB motif in the promoter of *SaCYP736A167* (a key santalol synthase gene) to promote sesquiterpenoid biosynthesis [11]. In *Salvia miltiorrhiza*, SmMYB36 positively regulates the expression of *SmDXS2* and *SmCPS1* to enhance tanshinones production [12], while SmNAC2 modulates multiple genes *KSL2*, *CYP76AH1*, *DXS2*, and *HMGR1* across both the MVA and MEP pathways to regulate tanshinones biosynthesis [13]. In *Phalaenopsis orchids*, the bHLH family member PbbHLH4 emerges as a master regulator of floral monoterpenes by activating the expression of *GERANYL DIPHOSPHATE SYNTHASE* (*PbGDPS*) [14]. In *Betula platyphylla*, BpWRKY6 integrates jasmonic acid (JA) signaling and terpenoid metabolism by directly binding to the promoters of JA biosynthesis genes (*BpLOX15*, *BpAOC4*) and the terpenoid synthase gene *BpCYP82G1*, thereby increasing total JA and terpenoid contents [15]. Despite these research advances across diverse species, the specific TFs and their crosstalk with JA that mediate terpenoid biosynthesis in oregano remain unexplored.

High-quality genomic data provide a critical foundation for understanding the genetics underlying terpenoid biosynthesis. Bornowski et al. [2] generated draft genome assemblies for four culinary herbs, including common oregano (*O. vulgare*), sweet basil (*Ocimum basilicum*), rosemary (*Rosmarinus officinalis*), and sweet marjoram (*O. majorana*), using the next-generation sequencing (NGS) technologies. The draft genome of *O. vulgare* spanned 630.04 Mb with a contig N50 of 157.94 kb [2]. Although this resources enable the identification of 33 terpene synthase (*TPS*) genes, its fragmented nature, specifically, the lack of chromosome-scale scaffolding, severely limits comprehensive studies of gene family evolution, synteny, and the regulation of biosynthetic pathways [2]. Compared to NGS technologies, third-generation sequencing (TGS) technologies such as PacBio SMRT and Oxford Nanopore sequencing, offer three core advantages: ultra-long read lengths, direct detection of epigenetic modifications, and single-molecule resolution for identifying rare variants [16]. These TGS technologies have been widely applied to generate high-quality genome assemblies for some medicinal plants including *Cornus wilsoniana* [ 17], *C. officinalis* [18], and *Mosla chinensis* [19]. However, a high-quality reference genome of Greek oregano is still needed for identifying key regulatory elements, gene family expansions, and structural variants associated with the production of bioactive terpenoids such as thymol and carvacrol.

A high-quality Greek oregano genome is essential to fill existing genomic gaps and lay a critical foundation for identifying key regulatory genes underlying the terpenoid biosynthesis. The objectives of this study were to: (i) construct a high-quality genome assembly of Greek oregano using TGS, (ii) clarify the evolutionary trajectory of oregano within the Lamiaceae family, and (iii) identify enzymatic genes and regulatory TFs involved in oregano terpenoid biosynthesis. Meanwhile, we identified high EO content Greek oregano mutants (abbreviated as HEO) from a γ-ray-induced mutant population. Integrated genomic and comparative transcriptomic analysis between HEO and wild-type (WT) oregano plants revealed the regulatory role of JA in terpenoid biosynthesis. We further demonstrated that a JA-inducible bHLH transcription factor, OvbHLH13, directly trans-activated *OvSDR1* to enhance thymol and carvacrol production. Beyond elucidating the evolutionary trajectory of oregano, the genome resources developed in this study provide breeders with valuable molecular information for the targeted improvement of oregano EO production.

## Results

### Genome sequencing, *de novo* assembly, and functional annotation

**Figure 1:**
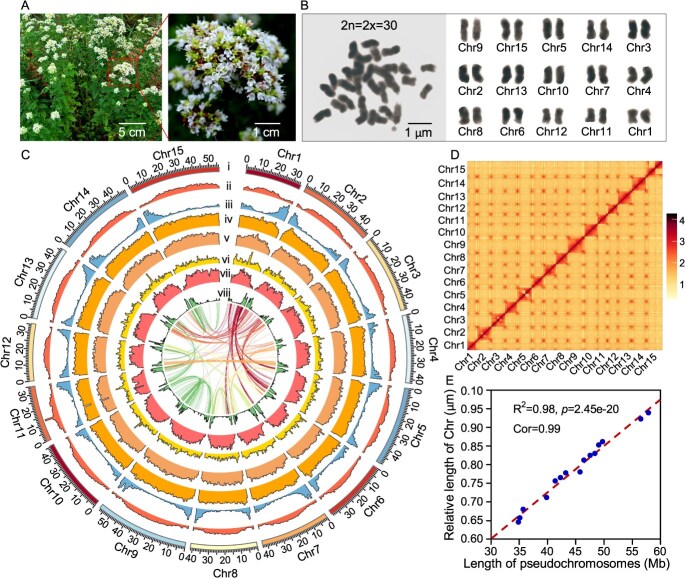
High-quality genome assembly and genomic features of *O*. *vulgare* subsp. *hirtum*. (A) Morphology of *O*. *vulgare* subsp. *hirtum* in the field depicting whole-plant architecture and floral morphology. (B) Karyotype analysis of Greek oregano. Individual chromosomes were digitally isolated and numbered based on the correlation between their observed physical lengths and assembly-derived lengths. (C) Genomic landscape across the 15 chromosomes. Scales for the chromosomes (i), GC content distribution (ii), gene density (iii), repetitive sequence density (iv), long terminal repeat (LTR) density (v), *Gypsy*-type LTR density (vi), *Copia*-type LTR density (vii), DNA transposable elements (TE) density (viii). (D) Scaffolding of 15 pseudochromosomes using Hi-C sequencing-based chromosome conformation capture data. (E) Linear regression analysis comparing the assembly lengths with the observed physical lengths of all chromosomes. Synteny blocks among 15 chromosomes were represented by the dotted lines.

Greek oregano, featured with white flower and high EO content, is a diploid species with 15 pairs of chromosomes (Fig. 1A and B). *K*-mer analysis estimated its genome size to be 732.76 Mb, with a heterozygosity rate of 1.58% and repetitive sequence content of 71.23% based on *k*-mer analysis (Fig. S1, Table S1, and Table S2). PacBio single-molecule real-time (SMRT) sequencing was performed to further improve genome assembly, yielding 796 contigs with a contig N50 of 25.75 Mb and a maximum contig length of 58.04 Mb (Table 1, Table S3, Fig. S2). These contigs were subsequently scaffolded using 79.00 Gb of Hi-C clean reads (Table S2, Table S4), anchoring 94.77% (672.60 Mb) of the total assembly into 15 pseudochromosomes. The final chromosome-scale assembly produced a total of 709.74 Mb, with a scaffold N50 of 46.36 Mb and chromosomes ranging from 34.90 to 57.79 Mb in length (Fig. 1C and D, Tables S5 and S6). Validation by comparison between assembled pseudochromosome lengths and measurements from somatic cells confirmed a high degree of consistency (Fig. 1E).

**Table 1:** The statistics of assembly and annotation of *O*. *vulgare* subsp. *hirtum* genome

Assembly and annotation features	Statistics
Illumina	
Estimated genome size	732.76 Mb
Heterozygosity	1.58%
Repeated content	71.23%
PacBio	
Assembly size	709.71 Mb
Contig N50 size	25.75 Mb
Contig N90 size Largest contig size	8.41 Mb
	58.04 Mb
GC content	40.22%
BUCSO for genome	96.20%
Hi-C assembly	
Assembly size	709.74 Mb
Number of scaffolds	781
Scaffold N50 size	46.36 Mb
Scaffold N90 size Largest scaffold size	34.90 Mb
	57.79 Mb
Chromosome anchored	94.77%
Annotation	
Number of protein coding gene	30 891
Average transcript length	2978 bp
Average coding sequence length	1175 bp
Percentage of repeat sequences	70.84%
Number of ncRNA	9579

To assess the completeness of the assembly and uniformity of sequencing coverage, Illumina short reads were mapped to the assembled genome. This evaluation demonstrated a high mapping rate of 98.94% and a genome coverage of 99.81% (Table S6). The assembly achieved a quality value (Qv) of 40.46, corresponding to a base accuracy exceeding 99.99% (Table S6). Further analysis of assembly completeness revealed that 96.20% of core genes were complete, with only 3.20% absent from the assembled genome (Fig. S3 and Table S7). Additionally, 232 out of 248 (93.55%) core genes were successfully identified within the assembly (Table S8). Collectively, the genome assembly of Greek oregano exhibited high accuracy, continuity, and comprehensive genome coverage.

The identified repetitive sequences spanned 502.77 Mb, constituting 70.84% of the genome (Table S9). Long terminal repeats (LTRs) were the predominant class, accounting for approximately 455.27 Mb (64.15% of the genome; Table S10). Within this category, Copia (319.02 Mb) and Gypsy (93.51 Mb) were the dominant LTR subtypes, comprising 44.95% and 13.18% of the whole genome, respectively (Table S11).

A total of 30 891 protein-coding genes were predicted (Fig. S4, Table S12), among which 29 457 genes were anchored to the 15 chromosomes (Chr) (Fig. 1C, Table S5), with the distribution ranging from 1665 genes on Chr11 to 2556 genes on Chr9 (Table S5). Gene copy number analysis revealed that 19 585 genes (63.40%) were present in multiple copies within the genome (Table S13). The average coding sequence lengths of all genes were 1175 bp, and the average transcript length was 2978 bp (Fig. S5, Table S14). Each gene contained an average of 4.71 exons, with a mean length of 249.44 bp (Table S14). Of all predicted genes, 98.23% were amenable to annotation using the SwissProt, KEGG, NR, InterPro, GO, and Pfam databases (Fig. S6; Table S15). In addition, non-coding RNA analysis revealed 4530 micro-RNAs, 5049 tRNAs, 6794 rRNAs, and 2503 snRNAs in the assembled genome of Greek oregano (Table S16).

As mentioned above, cytochrome P450 (CYP450) genes, particularly members of the *CYP71*, *CYP76*, *CYP706*, and *CYP736* subfamilies, are involved in the biosynthesis of major oregano EO constituents, including carvacrol, thymol, p-cymene, and thymohydroquinone [4]. In the Greek oregano genome assembly, we identified 302 *OvCYP* genes, which were grouped into 43 subfamilies (Fig. S7; Supplementary Material S1). Among these, 17 *CYP* genes belonging to the *CYP71*, *CYP76*, and *CYP706* subfamilies displayed differential expression between the HEO mutant and the WT plant (Fig. S7B), suggesting their potential roles in EO biosynthesis. Phylogenetic tree with known *CYPs* revealed candidate genes in the terpenoids biosynthesis: *evm.TU.Chr2.302*, *evm.TU.Chr3.1798*, *evm.TU.Chr9.202*, *evm.TU.Chr5.220*, and *evm.TU.Chr5.221* might be involved in monoterpenes synthesis; *evm.TU.Chr15.1472*, *evm.TU.Chr15.1473*, *evm.TU.Chr15.1474*, and *evm.TU.Chr15.2782* in sesquiterpene or monoterpene biosynthesis; and *evm.TU.Chr1.1655* in diterpene biosynthesis (Fig. S7C).

### Comparative genomics analysis

**Figure 2:**
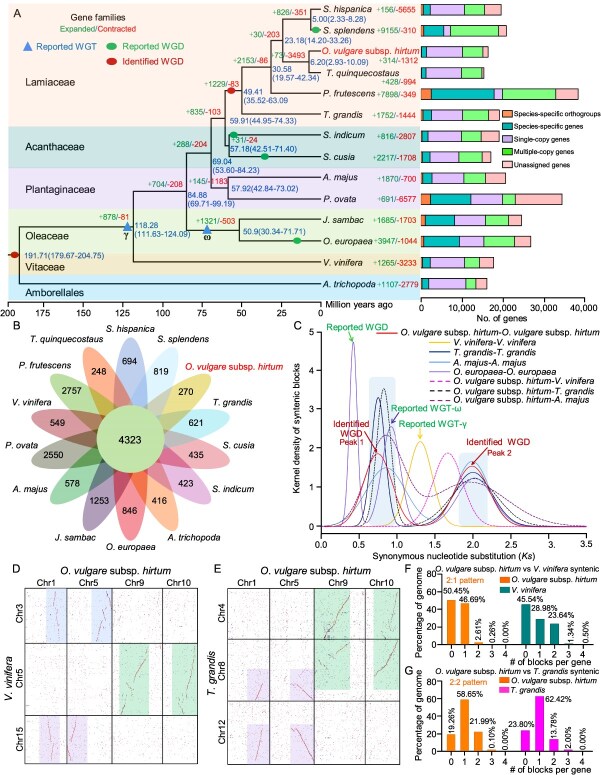
Phylogenetic analysis of *O*. *vulgare* subsp. *hirtum*. (A) The phylogenetic tree was constructed using the genomes of *O*. *vulgare* subsp. *hirtum* and 13 other plants. Genes of *O*. *vulgare* subsp. *hirtum* and other sequenced genomes were divided into five classes, including species-specific orthogroups, species-specific genes, single-copy genes, multiple-copy genes, and unassigned genes, with the numbers (n) of each class presented on the right side. (B) Venn diagram of the number of orthologous genes shared among the fourteen plants. (C) Distribution of average Ks between syntenic blocks. Peak 1 and Peak 2 correspond to two identified WGD events. (D, E) Syntenic block dotplot between *O*. *vulgare* subsp. *hirtum* and *V*. *vinifera* (D) or *O*. *vulgare* subsp. *hirtum* and *T*. *grandis* (E). (F, G) Ratio of synteny depth between *O*. *vulgare* subsp. *hirtum* and *V*. *vinifera* or *O*. *vulgare* subsp. *hirtum* and *T*. *grandis.*

A comparative genomics analysis was performed across 14 plant species to resolve the evolutionary placement of Greek oregano within the Lamiaceae (Fig. 2A, Table S17). The data spanned six representing diverse taxonomic orders, including Lamiaceae (*O*. *vulgare* subsp. *hirtum*, *Thymus quinquecostatus*, *Perilla frutescens*, *S. hispanica*, *S. splendens*, *Tectona grandis*), Acanthaceae (*Sesamum indicum* and *Strobilanthes cusia*), Plantaginaceae (*Antirrhinum majus* and *Plantago ovata*), Oleaceae (*Olea europaea* and *Jasminum sambac*), Vitaceae (*Vitis vinifera*), and Amborellales (*Amborella trichopoda*) (Fig. 2A, Table S17). Among them, *A*. *trichopoda*, an ANA-grade angiosperm species, was designated as outgroup. Phylogenetic analysis based on 431 orthologues indicated a close evolutionary relationship between *O*. *vulgare* subsp. *hirtum* and *T*. *quinquecostatus*, both members of the Lamiaceae, with an estimated divergence time of ~6.20 Mya (Fig. 2A). At a higher taxonomic level, Lamiaceae showed the closest affinity to Acanthaceae, followed Plantaginaceae and Oleaceae (Fig. 2A). It was estimated that the divergence between Lamiaceae and Acanthaceae occurred approximately 59.91 Mya (Fig. 2A). Comparative genome analysis across the 14 species identified 1312 gene families and 314 expanded gene families in Greek oregano (Fig. 2Aand B, Table S15, Material S2). KEGG and GO enrichment analysis revealed that the expanded gene families were primarily involved in functions such as serine/threonine-protein phosphatase activity, nonsense transcripts regulation, and secondary metabolic processes, etc. (Fig. S8). Furthermore, 270 gene families (comprising 837 genes) were specific to Greek oregano (Fig. 2C, Supplementary Material S3). KEGG analysis revealed that the top three enriched Greek oregano-specific gene families were FK506-binding nuclear protein, protein-lysine N-methyltransferase, and Zinc finger SWIM domain-containing protein 3 (Fig. S9).

### Karyotype evolution analysis

To investigate evolutionary divergence and whole-genome duplication (WGD) events, a comprehensive collinearity analysis was performed on *O*. *vulgare* subsp. *hirtum* and four representative species, including *T*. *grandis*, *A*. *majus*, *O*. *europaea*, and *V*. *vinifera*. The timing of WGD and speciation events in Greek oregano was predicated using the distribution of synonymous substitutions *per* synonymous site (Ks) among paralogous and orthologous genes. For Greek oregano, two distinct Ks peaks were observed at ~0.64 (peak 1) and ~1.98 (peak 2), corresponding to WGD events at ~58.92 and ~185.31 Mya, respectively (Fig. 2C). These WGD events were also identified in its closest relative *T*. *grandis*. Notably, the more recent WGD (~58.92 Mya) occurred after the well-characterized WGD in *V*. *vinifera* (~115.91 Mya), suggesting a lineage-specific duplication shared exclusively by *O*. *vulgare* subsp. *hirtum* and *T*. *grandis*. In contrast, the earlier WGD events (~185.31 Mya) predated the *V*. *vinifera* WGD, reflecting an ancient duplication conserved across five examined species. Given that *O*. *vulgare* subsp. *hirtum* and *T*. *grandis* diverged approximately ~6.20 Mya, the more recent WGD event likely occurred prior to their divergence (Fig. 2Aand C). Remarkably, *O*. *vulgare* subsp. *hirtum*, *T*. *grandis*, *A*. *majus*, and *O*. *europaea* all shared a WGD event at ~118.30 Mya, suggesting that this duplication predated the divergence of Lamiales species. An additional WGD event in *V*. *vinifera* was estimated at ~115.91 Mya (Fig. 2C), consistent with the previously reported gamma (γ) whole-genome triplication (WGT) event (~117.00 Mya) [20].

Furthermore, extensive chromosomal collinearity was observed at the whole-genome level among *O*. *vulgare* subsp. *hirtum*, *T*. *grandis*, and *V*. *vinifera* (Fig. 2Dand E). The homologous genes analysis identified a synteny ratio of 2:1 between oregano and *V*. *vinifera*, consistent with their established chromosomal collinearity (Figs 2Dand F and S10). In contrast, the comparation between *O*. *vulgare* subsp. *hirtum* and *T*. *grandis* revealed a distinct synteny ratio of 2:2. Specifically, 21.99% of *O*. *vulgare* subsp. *hirtum* genes were syntenic to a single genomic block in the *T. grandis* genome, while 13.78% of *T. grandis* genes corresponded to two blocks syntenic in the *O*. *vulgare* subsp. *hirtum* (Fig. 2E and G). Furthermore, the comparison of syntenic gene blocks between *O*. *vulgare* subsp. *hirtum* and *T*. *grandis* (*Ov*-*Tg*) revealed two Ks peaks that largely overlapped with those from *Ov*-*Ov* and *Tg*-*Tg*. This indicated that a high degree of chromosomal similarity had been retained since their divergence (Fig. 2C), which was consistent with their extensive collinear segments visible in the genome alignments (Fig. 2E and G).

**Figure 3:**
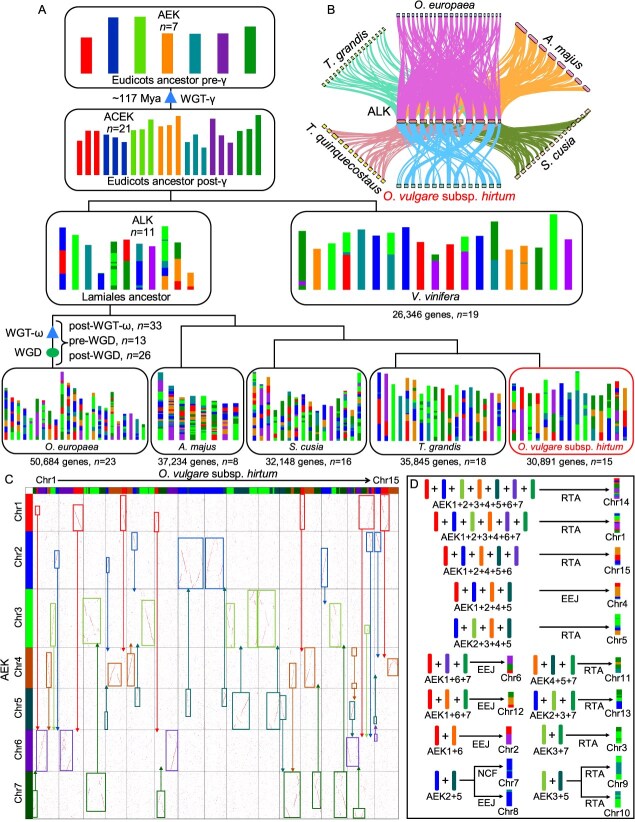
Evolutionary scenario of *O*. *vulgare* subsp. *hirtum* from the ancestral eudicot karyotype. Modern genomes were depicted at the bottom, with distinct colors indicating their derivation from seven ancestral chromosomes of the ancestral eudicot karyotype (AEK), ancestral core eudicot karyotype (ACEK), and ancestral Lamiales karyotype (ALK). (B) Collinear relationships among the ALK and six specific species. (C) Syntenic block dotplot between *O*. *vulgare* subsp. *hirtum* and ALK. (D) Ideograph of chromosome origin in Greek oregano.

To investigate the paleo-history of Greek oregano, we analyzed karyotype evolution across six species including *A*. *majus*, *O*. *europaea*, *S*. *cusia*, *T*. *grandis*, *V*. *vinifera*, and *O*. *vulgare* subsp. *hirtum*. The ancestral Lamiales karyotype (ALK) was reconstructed using *O*. *vulgare* subsp. *hirtum* and *T*. *grandis*, given their well-conserved genomic synteny (Fig. 3A). Following the WGT-γ event, the ancestral eudicot karyotype (AEK) descended to the ancestral core eudicot karyotype (ACEK), which comprised 21 protochromosomes (Fig. 3A) [17]. These 21 protochromosomes underwent a series of fusion and fission events, resulting in 11 chromosomes and distinct compositions in the ALK. Subsequently, the ALK evolved into *O*. *vulgare* subsp. *hirtum*, *A*. *majus*, *O*. *europaea*, *S*. *cusia*, and *T*. *grandis*, which experienced additional fusion and fission events (Fig. 3A). Accordingly, syntenic gene blocks were systematically identified between the ALK and six representative species (Fig. 3B). The protochromosome composition in *O*. *vulgare* subsp. *hirtum* became highly complex during speciation, with the evolutionary origins of distinct chromosomal segments traced in detail (Fig. 3C). In contrast, ALK and *V*. *vinifera* genomes served as controls, retaining intact protochromosomes. Furthermore, a clear 1:2 synteny ratio between the AEK and ALK was observed, implying that an additional WGD event occurred in Lamiales species following the WGT-γ event (Fig. S11).

To further elucidate chromosome evolution, the ideograph of chromosome origin in Greek oregano was drawn by linking oregano to the AEK through syntenic gene blocks, nested chromosome fusion (NCF), end-end joining (EEJ), and reciprocally translocated chromosome arms (RTAs) (Fig. 3D). For example, Chr14 was formed through an RTA event involving the first to seventh AEK protochromosomes, while Chr1 arose *via* RTA incorporating the first, second, third, fourth, sixth, and seventh protochromosomes. In contrast, Chr4 resulted from the EEJ-mediated fusion of first, second, fourth, and fifth AEK protochromosomes (Fig. 3D). The origins of Chr7 and Chr8 were inferred to involve NCF (from the second AEK protochromosome) and RTA (from the fifth AEK protochromosome), respectively (Fig. 3D). Similarly, Chr9 and Chr10 were both derived from RTA events involving the third and fifth AEK protochromosomes (Fig. 3D).

### Screening for Greek oregano mutants with high essential oil content

**Figure 4:**
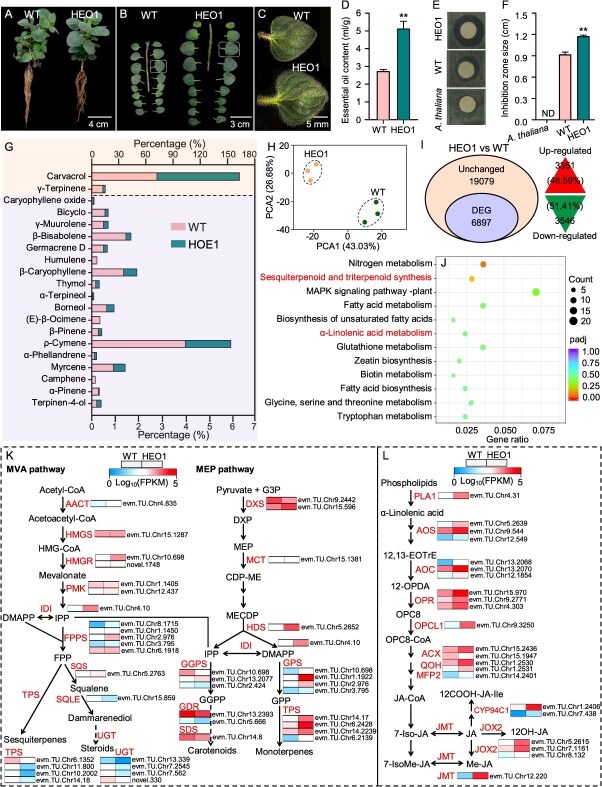
Morphological, antibacterial activity, and comparative transcriptomic analysis between the high essential oil mutant 1 (HEO1) and WT oregano. (A–F) Morphology, essential oil content, and antibacterial activity (shown as bacterial inhibition zone size) comparison between the HEO1 and WT oregano. Asterisks above bars indicate significant differences based on *p* values from the T-test assay (**for *p* < 0.01). Data were means ± SE (n = 4). (G) Essential oil compositions exhibiting statistically significant percentage differences between HEO1 and WT. (H) Principal component analysis (PCA) of the RNA-seq data. (I) Percentage of up- or down-regulated differentially expressed genes (DEGs) in ‘WT vs HEO1’. The numbers and percentages (in parentheses) of the DEGs are shown. (J) KEGG enrichment analysis of DEGs. (K) Expression profiles of enzymatic genes involved in the canonical MVA and MEP pathways for terpenoid biosynthesis. (L) Expression profiles of enzymatic genes in the linolenic acid metabolism pathway for JA biosynthesis.

Extracting EO from each plant could be extremely time-consuming and inefficient. Leveraging the antibacterial property of Greek oregano essential oil (EO), we established a rapid screening protocol based on its inhibitory effect of on *Escherichia coli* to identify mutants with higher EO yield (Fig. S12). The efficacy of the method was further verified by a correlation analysis between the areas of the bacterial inhibition zones and the EO contents of selected mutants (Fig. S12). A total of 51 mutants exhibited higher antibacterial properties than the WT plant (Fig. S13), which were classified as potential high essential oil mutants (HEOs). For example, HEO1, one representative mutant, had 5.12% EO, which was 88.24% higher than that of the WT (2.72%). Correspondingly, this mutant produced significantly larger bacterial inhibition zones (Fig. 4A-F). Furthermore, HEO1 had significantly larger leaf size, greater leaf thickness, and higher leaf-to-stem ratio than those of the WT (Figs 4 and S14). In addition to differences in EO yield, the EO composition also varied between HEO1 and WT, with HEO1 having significantly higher carvacrol but lower γ-terpinene (the precursor of carvacrol) contents than WT (Fig. 4G). Chromosome counting conformed that HEO1 maintained the same chromosome number as the WT (2*n* = 2*x* = 30, Figs 1B and S15).

### Involvement of JA in Greek oregano EO production

A comparative transcriptomic analysis between HEO1 and WT plants identified 6897 differentially expressed genes (DEGs), of which 48.59% were up-regulated, and 51.41% were down-regulated (Fig. 4H and I). Principal component analysis (PCA) confirmed that samples from the same group exhibited similar gene expression patterns. The reliability of the RNA-seq data was further validated by qRT-PCR analysis of nine randomly selected DEGs, which showed a strong correlation with the transcriptome data (correlation coefficient *R*^2^ = 0.95) (Figs 4H and I and S16). Further KEGG enrichment analysis revealed that DEGs were primarily enriched in pathways including sesquiterpenoid and triterpenoid biosynthesis, α-linolenic acid metabolism, tryptophan metabolism, fatty acid metabolism, biosynthesis of unsaturated fatty acids, MAPK signaling pathway, glutathione metabolism, and zeatin biosynthesis. (Fig. 4J). In plants, terpene biosynthesis was mediated by two canonical pathways: the mevalonic acid (MVA) and the methylerythritol phosphate (MEP or non-mevalonate) pathways [21]. Eighteen out of 22 genes of the MVP pathway and 18 out of 19 genes of the MEP pathway were differentially expressed between WT and HEO1. Specifically, 10 out of 11 genes involved in monoterpenes biosynthesis (from MEP to monoterpenes) in the MEP pathway showed significantly higher expression in HEO, which aligned with the elevated contents of carvacrol and total EO observed in HEO1 (Fig. 4D and K). Additionally, 21 out of 22 genes in the α-linolenic acid metabolism involving the JA biosynthesis pathway had significantly higher expression levels in HEO1 (Fig. 4L), suggesting that enhanced JA biosynthesis might be positively associated with the increased terpenoid production in Greek oregano.

**Figure 5:**
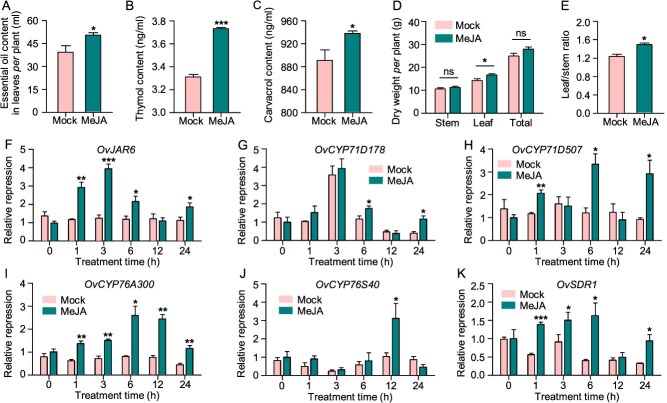
Exogenous JA application increased EO yield and activated the expression of key EO biosynthetic genes in Greek oregano. (A-E) Effects of exogenous application of JA on EO content (A), thymol content (B), carvacrol content (C), dry weight (D), and leaf to stem ratio (E) in Greek oregano. (F-J) Effects of exogenous application of JA on relative expression of key JA signaling gene (*OvJAR6*) and key EO biosynthetic genes, including *OvCYP71D178*, *OvCYP71D507*, *OvCYP76A300*, *OvCYP76S40*, and *OvSDR1.* Asterisks above bars indicate significant differences based on *p* values from the T-test assay (* for *p* < 0.05; **for *p* < 0.01; *** for *p* < 0.001). Data are means ± SE (n = 3 in A-J).

To verify whether JA plays a role in regulating EO biosynthesis, we sprayed JA on Greek oregano. This application led to a 27.95% increase in the EO yield, including 12.69% higher thymol content and 5.28% higher carvacrol content (Fig. 5A–C). Concomitantly, JA treatment also enhanced biomass traits, increasing leaf weight by 16.73% and leaf-to-stem ratio by 20.80% (Fig. 5Dand E). At the molecular level, JA application significantly upregulated expression of the EO signaling gene (*OvJAR6*) and biosynthetic genes, including *OvSDR1*, *OvCYP71D178*, *OvCYP71D507*, *OvCYP76A300*, and *OvCYP76S40* that whose encoded enzymes catalyze the formation of thymol and carvacrol from GDP (Fig. 5F-K). These coordinated phenotypic and transcriptional responses indicated that JA positively regulated terpenoid biosynthesis in Greek oregano.

### Identification of a JA-inducible bHLH transcription factor activating the expression of the terpenoid-biosynthesis gene, *OvSDR1*

**Figure 6:**
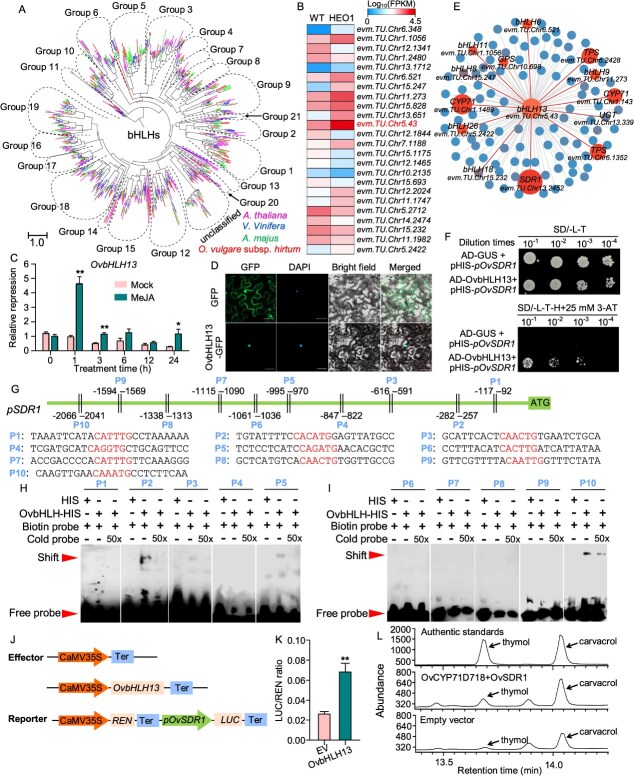
A JA-inducible transcription factor, OvbHLH13, directly transactivated *OvSDR1* in the terpenoid biosynthesis pathway. (A) Phylogeny of *bHLHs* identified in *O*. *vulgare* subsp. *hirtum*, *A*. *thaliana*, *V*. *Vinifera*, and *A*. *majus* genomes. (B) Expression profiles of the differentially expressed Greek oregano *bHLHs*. *evm.TU.Chr5.43* is the gene ID of *OvbHLH13*. (C) Relative expression analysis of *OvbHLH13* with JA treatment. (D) Subcellular localization of OvbHLH13. (E) Co-expression network analysis was built using differentially expressed genes in major terpenoid biosynthesis pathways. (F) Y1H assay*.* (G-I) EMSA assay*.* There are ten candidate *cis*-elements on the *OvSDR1* promoter, designated as P1-P10, which were used as probes in the EMSA. (J&K) Dual-luciferase assay*.* (L) GC–MS chromatograms of products from the transient transformation of *OvSDR1* in *N. benthamiana* with the substrate γ-terpinene. Enzyme products were identified by comparing their retention times and mass spectra to those of authentic standards. Data in (C&K) were means ± SD (n = 3 in C and n = 6 in K). Asterisks above bars in (C&K) indicated significant differences based on *p* values from the T-test assay (* for *p*<0.05; **for *p*<0.01).

Basic helix–loop–helix (bHLH) transcription factors play a crucial role in JA signaling and secondary metabolism. In this study, a total of 146 bHLH transcription factors were identified in the Greek oregano genome, which were classified into 21 groups (Fig. 6A, Material S4). Among these, 24 *bHLH* genes were differentially expressed between HEO1 and WT plants (Fig. 6B). Of them, *evm.TU.Chr5.43* (named as *OvbHLH13*; Material S3), exhibited the highest expression level in HEO1 (Fig. 6B). Its expression increased more than 4-fold after 1 hour of JA treatment (Fig. 6C), suggesting its involvement in JA-mediated regulation of terpenoid biosynthesis. By transiently expressing *OvbHLH13-GFP* in *Nicotiana benthamiana,* the GFP signal of the OvbHLH13-GFP fusion protein was exclusively localized to the nucleus (Fig. 6D).

To identify the target genes of OvbHLH13, we performed a co-expression network incorporating key terpenoid biosynthesis genes including *CYPs*, MEP/MVA pathway genes and other *OvbHLH* genes (Fig. 6E). The results revealed that *OvbHLH13* was significantly co-expressed with *OvSDR1*, *OvCYP76S40* (*evm.TU.Chr5.220* and *evm.TU.Chr5.221*), *OvGPS* (*evm.TU.Chr10.698*), and *OvTPS1.1&3.1* (*evm.TU.Chr6.1352* and *evm.TU.Chr6.2428*) (Fig. 6E). The bHLH domains of bHLH transcription factors bound to the E-box (CANNTG with N = A/C/G/T) or the canonical G-box (CACGTG) *cis*-elements [22]. Therefore, the co-expressed genes identified here are promising candidates for direct regulation by *OvbHLH13* through such promoter elements. Therefore, we carried out an *in silico* promoter analysis of these associated genes. The promoter of *OvSDR1* contained ten E-box *cis*-elements potentially recognized by bHLH transcription factors (Fig. 6G). A multi-assay approach was then employed to validate this direct regulation. Firstly, a yeast one-hybrid (Y1H) assay confirmed that OvbHLH13 bound to the promoter of *OvSDR1* (Fig. 6F). Second, an electrophoretic mobility shift assay (EMSA) demonstrated the purified OsbHLH13 protein could directly bind to DNA sequences flanking the E-box *cis*-elements in the *OsSDR1* promoter *in vitro* (Fig. 6G). The EMSA result showed that OvbHLH13 bound to P2, P3, P5, and P10 probes, with a strongest binding affinity observed for the P10 probe (Fig. 6Hand I). Third, an *in planta* transactivation assay in *N. benthamiana* showed that co-expressing *35S::OvbHLH13* with *OsSDR1-Pro::LUC* with *35S::REN* as the internal control led to a significant increase in luciferase activity (LUC/REN ratio), indicating that OvbHLH13 activated *OvSDR1* expression in living plant cells (Fig. 6J and K).

Previous studies have demonstrated that cytochrome P450 enzymes (CYP450s), including TvCYP71D178 and TvCYP71D507, catalyze the hydroxylation of γ-terpinene at either the C-3 or C-6 position. These reactions produce dienol intermediates, which were subsequently converted by TvSDR1 into thymol and carvacrol in *T. vulgaris* [4]. In this study, co-overexpression of *OvCYP71D178* and *OvSDR1* in *N. benthamiana* significantly enhanced the production of thymol and carvacrol, two major bioactive monoterpenoids in *O. vulgare* subsp. *hirtum* EO. Together, these results confirmed that bHLH13 was an important transcription factor that directly transactivated the expression of *OvSDR1*, thereby regulating the biosynthesis of EO in Greek oregano.

## Discussion

The present study reports a high-quality, chromosome-level genome assembly of Greek oregano (*O*. *vulgare* subsp. *hirtum*), an economically important subspecies for its EO rich in terpenoids such as carvacrol and thymol [3–5]. Notably, oregano EO is a high-value commercial product with demonstrated diverse bioactivities, including antioxidant, anti-inflammatory, antiangiogenic, anticancer, and antimicrobial properties [23]. Through integrating genomic, transcriptomic, and functional validation approaches, we not only elucidated the evolutionary trajectory of Greek oregano and its influences on terpenoid biosynthesis, but also uncovered a JA-mediated regulatory module centered on the transcription factor OvbHLH13, which critically governed terpenoid biosynthesis. These findings provide a foundational genomic resource for advancing our understanding of aromatic plant biology and accelerating molecular breeding of Greek oregano and related species within Lamiaceae family.

### A high-quality genome assembly of Greek oregano filled key genomic gaps

Previous attempts to sequence the oregano genome using NGS technologies produced a fragmented assembly with a contig N50 of only 157.94 kb and no chromosomal anchoring, thus limiting its uses in identifying structural variations, syntenic relationships, and gene regulatory networks associated with terpenoid biosynthesis [2]. Here, we presented a high-quality genome assembly of *O. vulgare* subsp. *hirtum*, integrating PacBio SMRT long-read sequencing with Hi-C scaffolding, and constructed 15 pseudochromosomes (spanning 709.74 Mb) with a scaffold N50 of 46.36 Mb. This assembly demonstrated high continuity (94.77% of sequences anchored to chromosomes), accuracy (Qv = 40.46, >99.99% base accuracy), and completeness (96.20% of conserved eukaryotic genes recovered). Collectively, this genome assembly represents a substantial improvement over previous resources and provides a robust genomic framework for future evolutionary, comparative, and functional studies in Greek oregano and related Lamiaceae species.

Notably, the genome annotation of 30 891 protein-coding genes, including 302 *cytochrome P450* (*CYP*) genes (43 subfamilies), provides a comprehensive catalog of candidate genes involved in terpenoid metabolism. Among these *CYPs*, 17 members of the *CYP71*, *CYP76*, and *CYP706* subfamilies, known to regulate carvacrol, thymol, and p-cymene biosynthesis in oregano and related species (e.g., thyme), exhibited differential expression between the HEO1 mutant and WT plants. Phylogenetic analysis further suggested that several of these *CYPs* (e.g., *evm.TU.Chr2.302*, *evm.TU.Chr3.1798*) might contribute to terpenoid biosynthesis, highlighting the value of this genome assembly for prioritizing uncharacterized genes for functional validation. Collectively, this high-quality genomic resource fills a long-standing and critical gap for Lamiaceae genomics, enabling comparative analyses across aromatic species and facilitating the dissection of genetic mechanisms underlying terpenoid production.

### Whole genome duplication and karyotype evolution shaped terpenoid biosynthesis in Lamiaceae

The chromosome-level genome assembly of Greek Oregano provided a foundation for investigating genome evolution and gene family expansion in Lamiaceae [23]. Using the genome data, we were able to reconstruct the evolutionary trajectory across Lamiales, the Lamiaceae family, and within oregano itself. Polyploidy plays a pivotal role in plant evolution and functional innovation [24, 25]. Lamiales experienced the core eudicot-wide WGT-γ event, which occurred ~115–130 Mya [26]. In the present study, we identified a WGD event in *V*. *vinifera* at ~115.91 Mya (Fig. 2C), consistent with the previously results [20, 26]. Subsequently, we found that Greek oregano and *T*. *grandis* (Lamiaceae), *A*. *majus* (Plantaginaceae), and *O*. *europaea* (Oleaceae) all shared a WGD event at ~115.91 Mya, suggesting that this ancient WGD event occurred prior to the divergence of these major Lamiales species. This finding aligned with a recent reporting that *C. americana* and *T. grandis* underwent two polyploidization events: a core WGT-γ event and a later WGD-β event shared among Lamiales families, including Scrophulariaceae, Acanthaceae, Bignoniaceae, and Lamiaceae [27]. The detection of a shared WGD event between Lamiaceae and *V*. *vinifera* (~115.91 Mya) aligned with the proposed WGT-γ (~117.00 Mya) event in core eudicots [20], indicating that such duplication played a role in driving the diversification of terpenoid biosynthetic pathways within Lamiaceae. WGD events are known to drive the expansion of gene families involved in secondary metabolism, as duplicated genes can acquire novel functions or partition ancestral roles [28]. In Greek oregano, the expanded gene families, enriched in secondary metabolic processes, likely include key genes in terpenoid biosynthesis (e.g., *TPS*, *CYP*, *SDR*), thereby providing a genetic basis for its high EO content. Notably, the expansion of specific gene families such as *CYP450* and *bHLH* in Greek oregano (314 expanded families, 270 specific orthologues) correlated with enhanced metabolic plasticity, particularly in pathways linked to secondary metabolism. Collectively, these results underscore the significant role of WGD in shaping the adaptive evolutionary processes of secondary metabolism among aromatic plants [27].

Karyotype evolution analysis further revealed the dynamic origins of Greek oregano’s 15 chromosomes from the ancestral eudicot karyotype (AEK). Through nested chromosome fusion (NCF), end-end joining (EEJ), and reciprocal translocation of chromosome arms (RTA) [17], the 21 protochromosomes of the ancestral core eudicot karyotype (ACEK) were reduced to 11 in the ancestral Lamiales karyotype (ALK), and subsequently to 15 in Greek oregano. This structural reorganization likely led to the clustering of terpenoid biosynthetic genes, an observation supported by syntenic analysis that revealed colocalization of *CYP*, *TPS*, and *SDR* genes on specific chromosomes (e.g., Chr5 and Chr6). Gene clustering is a common feature of secondary metabolic pathways, facilitating coordinated regulation and efficient metabolite production [29]. For example, on chromosome 5, the transcription factor of *OvbHLH13* was colocalized with its putative target genes *OvSQS*, *OvGDR*, and *OvHDS* potentially forming a tightly regulated transcriptional control of thymol/carvacrol biosynthesis (Fig. 4K), highlighting the role of karyotype evolution in shaping regulatory networks.

### An oregano mutant population enables the discovery of terpenoid biosynthetic regulatory pathways

While the established mutant population with varied EO profiles is a valuable resource for elucidating terpenoid biosynthesis [2, 4], the comprehensive chemical characterization of mutants is laborious and time-consuming. Taking advantage of the strong antimicrobial properties of oregano EO [1, 3], we developed a rapid screening method to select Greek oregano mutants with high antibacterial inhibition effect. In total, 51 mutants exhibited enhanced antibacterial activities compared to the WT, which holds potential for oregano breeding or development as new varieties via vegetative propagation methods, such as cuttings or micropropagation. HEO1 mutant was distinguished by its exceptional antibacterial properties and a markedly elevated EO content (5.12% vs. 2.72% in WT). Consequently, it was selected for further transcriptome analysis.

It is interesting to point out that HEO1 had a normal chromosome number (2*n* = 2*x* = 30, identical to WT), confirming that γ-ray-induced genetic variations in this mutant did not change ploidy level. Instead, it likely resulted from single-base substitutions (e.g., transition/transversion), Indels, large DNA fragment rearrangement, and hypomethylation, all consequences of damaging DNA bases and creating double-strand breaks (DSBs) [30, 31]. Such mutations in coding regions or *cis*-regulatory regions of terpenoid-regulatory genes (e.g., *OvSDR1*, *OvbHLH13*, *OvCYP71D178*, etc.) involved in the MVA/MEP pathway might increase their expression levels, enhance the chromatin accessibility, stabilize their mRNAs, or increase protein accumulation. Consistent with this model, the upregulated key MVA/MEP pathway genes correlated directly with the higher total EO yield of the HEO1 mutant. Future studies are needed to clarify the exact mutation mechanism of HEO1, ultimately providing deeper insights into terpenoid biosynthesis for informing precision breeding strategies for aromatic plant breeding.

### JA is crucial in the regulation of terpenoid biosynthesis

JA-mediated induction of terpenoid biosynthesis was well-documented [32, 33]. For example, in *Mentha arvensis*, MeJA treatment increased terpenoid content and upregulated the expression of JA signaling genes (*JAZs*, *MYCs*) and monoterpenoid biosynthetic genes (e.g., *GPPSs*, *LSs*) [33]. In *Betula platyphylla*, BpWRKY6 integrates JA signaling and terpenoid metabolism by directly binding to the promoters of JA biosynthesis genes (*BpLOX15*, *BpAOC4*) and the terpenoid synthase gene *BpCYP82G1*, thereby increasing contents of total JA and terpenoid [15]. In Greek oregano, we validated and extended this conserved regulatory module. Exogenous JA application significantly increased EO content by 27.95% increase in EO content and thymol by 12.69%, and coordinately upregulated key EO biosynthetic genes (e.g., *OvSDR1*, *OvCYP71D178*, and *OvCYP71D507*).

The integration of transcriptomic data from HEO1 and WT plants, combined with exogenous JA treatment, uncovered a pivotal role for JA in regulating terpenoid biosynthesis in Greek oregano. Comparative transcriptomic profiling identified 6897 differentially expressed genes (DEGs), with significant enrichment in pathways including α-linolenic acid metabolism (JA biosynthesis), sesquiterpenoid/triterpenoid biosynthesis, and MAPK signaling. Notably, 21 of 22 genes in the JA biosynthetic pathway were up-regulated in HEO1, along with increased expression of 10 out of 11 monoterpenoid biosynthetic genes in the methylerythritol phosphate (MEP) pathway, consistent with the higher carvacrol content and total EO yield of HEO1.

### OvbHLH13 is a JA-inducible transcription factor regulating the expression of *OvSDR1* in terpenoid biosynthesis

Through integrated genomic and transcriptomic analysis, we identified a key transcription factor in Greek oregano, *OvbHLH13*, as the core gene connecting DEGs across major terpenoid biosynthesis pathways, including the MVA, MEP, α-linolenic acid metabolism pathway, *CYP*, *TPS*, and *bHLH* family genes. The transcription of *OvbHLH13* was specifically induced by JA, indicating its role in the regulation of terpenoid biosynthesis. bHLH family members were known to mediate JA signaling and secondary metabolism in plants [14, 22, 34, 35]. For instance, in *Arabidopsis thaliana*, bHLH transcription factors GL3 and EGL3 interact with MYB75 to form regulatory complexes that coordinate JA-mediated anthocyanin accumulation and trichome initiation [34]. Similarly, in *Dendrobium officinale*, the MeJA-responsive DobHLH4 transcription factor directly activates *DoTPS10*, a key gene for linalool biosynthesis during floral development [35].

Based on the predicted DNA-binding site of the bHLH transcription factor, we identified a potential direct downstream gene, *OvSDR1*. Subsequent experimental assays confirmed that OvbHLH13 directly activated the transcription of *OvSDR1* but not downstream *CYPs*. As mentioned previously, OvSDR1 was an orthologue to TvSDR1 in thyme that dehydrogenates cyclohexadienol intermediates to produce precursors of thymol and carvacrol [4]. This regulatory roles of bHLH in secondary metabolism have been supported by findings in other plants. For example, in *Artemisia annua*, AabHLH1 enhanced artemisinin biosynthesis by activating *amorpha-4,11-diene synthase* [36]. Similarly, in *S. miltiorrhiza*, SmNAC2 modulated tanshinone synthesis *via HMGR1* and *CYP76AH1* [13]. The results established an ‘OvbHLH13-*OvSDR1*’ regulatory module that could be used to enhance the production of oregano terpenoid.

In summary, we generated a high-quality genome assembly of Greek oregano, providing a crucial resource to elucidate the evolutionary trajectory of the Lamiales, the Lamiaceae family, and oregano itself. This assembly enabled the identification of species-specific and expanded genes. We further pinpointed a core transcription factor, OvbHLH13, which formed a regulatory module with *OvSDR1* to govern terpenoid biosynthesis. Through a rapid screening method, we selected a Greek oregano mutant that nearly doubled the EO yield of WT plant. This genomic resource and identified candidate genes paved the way for further optimizing terpenoid biosynthetic flux in breeding programs for Greek oregano and other related aromatic species.

## Materials and Methods

### Plant materials and screening for high EO content Greek oregano mutants

The Greek oregano for genome sequencing was obtained from the USDA-ARS Germplasm Resources Information Network (GRIN accession No.: PI 384485). To induce genetic variation， seeds harvested from the maternal plants, were exposed to ^60^Co-γ radiation at a dose of 100 Gy (Fig. S12). The treated seeds were germinated in trays in a greenhouse. Upon reaching approximately 8 cm in height, a total of 8170 seedlings were transplanted to the experimental field at the Nanjing Agricultural University Lishui Campus (Nanjing, China) for subsequent screening and analysis.

For rapid screening of the oregano mutants with high antibacterial effects and potentially high EO content, five leaf discs (6 mm in diameter) *per* plant were collected from each of 8170 individual Greek oregano plants at the stage of full blossom with a hole puncher. The discs were placed into 1.5-ml centrifuge tubes, homogenized in 1 ml dimethyl sulfoxide (DMSO), and incubated overnight at 25°C. Then, the mixture was centrifuged at 5000 rpm for 5 minutes, and the supernatant was retained as leaf extracts (containing oregano EO). Paper discs (6 mm in diameter) were immersed in the leaf extract and then placed onto an LB medium plate freshly with inoculated *E. coli* strain ‘T1’. After 24 hours of incubation, the diameter of the resulting bacterial inhibition zones was clearly visible and measured using Digimizer software version 5.7.2. (https://www.digimizer.com). Mutants with obviously larger bacterial inhibition zones than the maternal plants (WT) were selected for further EO content analysis using the traditional EO distillation method as described below.

### Genome sequencing, assembly, and annotation

Genome sequencing was performed by Novogene Co., Ltd. (Tianjin, China) using a multi-platform strategy: Illumina-based sequencing, PacBio sequencing, and Hi-C sequencing. Genome size and heterozygosity were estimated using *k*-mer analysis of Illumina sequence data with Jellyfish v2.2.7 [37] and GenomeScope v1.0 [38]. Clean reads from PacBio sequencing were assembled into contigs with the Hifiasm-v0.16.1 program under default parameters. A haplotype-aware error correction was initially conducted using Hifiasm-v0.16.1 [39] to rectify sequence errors while preserving heterozygous alleles. Then, a phased assembly graph was constructed utilizing local phasing information derived from the corrected reads.

For the integration of structural information derived from Hi-C data, the Hi-C clean reads were first aligned to the draft scaffold assembly using BWA-mem v0.7.8 [40]. The assembly and scaffolds orientation for the 15 Greek oregano chromosomes were performed with LACHESIS v210701 [41] and Juicebox v1.11.08 [42]. The interaction patterns within chromosomes were visualized *via* a Hi-C matrix heat map generated with HiCExplorer 3.7.230 [43]. Gene structural annotation was predicted using three methods: *ab initio* prediction, homology-based prediction, and RNA-Seq assisted prediction. Gene and protein functions were annotated by aligning sequences to the Swiss-Prot database and the NR database.

### Phylogenetic analysis

The evolutionary connections between Greek oregano and fourteen other plant species, with *V*. *vinifera* set as the out-group, were investigated (Table S17). A phylogenetic tree was constructed using single-copy orthologous genes identified at the whole-genome level. Single-copy orthologous gene families (orthogroups) with complete species coverage were then identified using OrthoFinder [44], and their coding sequences (CDS) were extracted for analysis. Sequence alignment was performed using MAFFT [45] with default parameters. Subsequently, sequences were trimmed using the ‘automated1’ mode in trimAl [46]. A maximum likelihood tree was constructed from the trimmed alignments with RAxML [47] using the ‘GTR + GAMMA’ model, and branch support was assessed with 100 bootstrap replicates. Finally, the gene tree information was integrated into a consensus species tree using ASTRAL-III [48]. This entire analysis pipeline was implemented using PhyloForge [49].

### Divergence time estimation and gene family expansion

The molecular clock model, implemented in the MCMCtree module of PAML [50], was employed to estimate the divergence times between species. Calibration points for the analysis were obtained from the Timetree database (https://timetree.org/), including (i) the divergence between *A. trichopoda* and core angiosperms (179.9–205 MYA) and (ii) the divergence between *P*. *frutescens* and *T*. *grandis* (33.3–72.4 MYA). To ensure statistical reliability, the convergence result was assessed using Tracer [51], requiring an effective sample size (ESS) >200 for all parameters.

Gene family expansion analysis was carried out with CAFE5 [52] using the ‘-k 6’ parameter. The gene family matrix generated by OrthoFinder and the time-calibrated species tree were used as input. The results were visualized using the CafePlotter software. Based on OrthoFinder clustering results, the core gene families and species-specific gene families were visualized in R software v4.0.2.

### Genome synteny and WGD analysis

WGD events were predicted based on analysis of synteny and the synonymous substitution rate (Ks) value. Within the oregano genome, intraspecific homologous gene pairs were identified using BLASTP with an *E*-value cutoff set to ≤1e-5. WGDI [53] was used to generate a whole-genome dot plot using the -d parameter, from which syntenic blocks were extracted using the -icl parameter. The Ks values of homologous gene pairs were computed, and the Ks peak was fitted (−pf parameter) to estimate the timing of WGD events. Cross-species synteny analysis was performed using JCVI [54] to validate the identified polyploidy events within a broader evolutionary context.

Leveraging the Ancestral Eudicot Karyotype model (AEK, *n* = 7) proposed by Wang et al. [55] serving as a reference, we utilized the karyotype mapping module (−km parameter) of WGDI to align ancestral chromosome segments with the genomes of extant species. This analysis allowed chromosome rearrangement events to be identified, including fusions and fragmentations. Key parameters and command-line implementations for some software tools are listed in Supplementary Material S6.

### Identification and characterization of bHLH and CYP family members

Homologous CYPs in Greek oregano were predicted using BLASTP with an *E*-value cutoff set to ≤1e-5. Sequences with an identity percentage greater than 40.00% were retained for further analysis. The HMMER software [56] was used to identify domains using the Hmmscan program with an E-value cutoff set to ≤1e-5. Sequences containing the HMM (PF00067) model were considered as candidate OvCYPs. Phylogenetic trees for AtCYPs and OvCYPs were constructed with IQ-TREE using the aligned and trimmed sequences [57]. The Greek oregano OvCYPs were classified into subfamilies based on their clustering with of *Arabidopsis* AtCYPs in the phylogenetic tree.

For *O*. *vulgare* subsp. *hirtum*, *V*. *Vinifera*, and *A*. *majus*, proteins containing the HMM (PF00010) domain were selected as candidate bHLHs. Phylogenetic trees for the bHLHs in *O*. *vulgare* subsp. *hirtum*, *A*. *thaliana*, *V*. *Vinifera*, and *A*. *majus* were constructed using the aligned and trimmed sequences with IQ-TREE [57]. The OvbHLHs were classified based on both scheme established by Toledo-Ortiz et al. [58] and the constructed phylogenetic tree.

### Differentially expressed gene analysis

For RNA-seq analysis, cDNA libraries were constructed and sequenced using the Illumina platform, according to the standard manufacturer’s protocol (Illumina, San Diego, CA, USA). Differentially expressed genes were identified using a fold change (FPKMa/FPKMb) > 2 and a false discovery rate (FDR) < 0.01. The relative expressions of candidate genes were further validated by qRT-PCR assay, using *OvEF1a* as the reference gene [4].

### Essential oil yield and composition analyses

The EO was extracted using a distillation extraction method. Briefly, Greek oregano leaves and/or flowers were harvested, shade-dried to a stable weight, and then placed in the distillation apparatus containing 3 L of deionized water. The mixture was heated to 100°C and maintained at boiling for 90 min. The EO content was calculated using the following formula: EO yield (%) = (yield of EO/dry matter of samples) × 100. The chemical components of Greek oregano EO was measured by gas chromatography–mass spectrometry (GC–MS, 7890A-5975C, Agilent) according to the protocol described by Bornowski et al. [2]. In addition, semi-quantitative analysis of thymol and carvacrol contents in the EO was conducted by GC–MS using authentic standards of thymol and carvacrol at known concentrations for calibration.

### Subcellular localization analysis of OvbHLH13

The coding sequence (CDS) of *OvbHLH13* was cloned into the pEarleyGate103 vector to generate *OvbHLH13*-*GFP* fusion constructs. The resulting construct was introduced into *Agrobacterium tumefaciens* strain ‘EHA105’. *Agrobacterium* cultures harboring the pEarleyGate103-*OvbHLH13* were infiltrated into *N. benthamiana* leaves according to the method described by Zhang et al. [59]. After 2–3 days of *Agrobacterium*-infiltration, leaves were collected for microscopic examination to detect GFP fluorescence signals. Nucleus was stained with DAPI as a control.

### Y1H and dual-LUC reporter assay

The CDS of *OvbHLH13* and the promoter sequence of *OvSDR1* were inserted into pGADT7 and pHis2.1 vectors, respectively. These constructed vectors were co-transformed into the yeast strain ‘Y187’, which was subsequently screened on the yeast synthetic dropout SD/-Trp-Leu-His medium. This medium was supplemented with 25 mM 3-AT.

The CDS of *OvbHLH13* was cloned into the pGreenII-62SK vector to create the effector construct. Meanwhile, the promoter sequence of *OvSDR1* was inserted into the pGreenII-0800 vector to generate the reporter construct. Both constructs (effector and reporter) were co-expressed in leaves of *N. benthamiana*. Leaf samples were collected 48 hours after *Agrobacterium*-infiltration, followed by the analysis of firefly luciferase (LUC) and renilla luciferase (REN) activities. Promoter activity was expressed as the ratio of LUC to REN activity.

### Electrophoretic mobility shift assay

The OvbHLH13 protein could not be expressed efficiently in prokaryotic systems, thus requiring codon optimization. The optimized gene sequence is provided in Material S5. The codon-optimized *OvbHLH13* was inserted into the pET30a-1 vector and then transformed into cells of *E. coli* strain ‘BL21’. BL21 cells harboring pET30a-1-*OvbHLH13* was grown in LB liquid medium supplemented with 0.5 mM IPTG as the protein expression inducer. Both the GST protein and the recombinant protein were purified for EMSA. The biotin-labeled probe and corresponding unlabeled cold probe were synthesized by General Biosystems (Hefei, China). Primer sequences for EMSA were provided in Table S18.

### Enzymatic analysis of OvSDR1 in terpenoid biosynthesis

*OvCYP71D178* and *OvSDR1* were cloned into pEarleyGate103 and co-transformed into leaves of *N. benthamiana*. Following transformation, transformed plants were kept in darkness for 12 hours and then transferred to light for 3 days. Ten leaf discs (10 mm in diameter) were collected from the infiltrated areas using a cork borer and submerged in 20 mM citrate–phosphate buffer (pH 7.4) containing 4 mM γ-terpinene. The leaf discs were vacuum-infiltrated for 20 minutes to facilitate substrate uptake and then incubated under light at room temperature for 24 hours. Following incubation, the leaf discs were homogenized in 1 ml of methanol and incubated at −20°C for 16 hours. After centrifugation at 5500 g for 20 minutes, a 200 μl aliquot of supernatant was analyzed by GC–MS using thymol and carvacrol standards (0.1 μg/ml each).

## Supplementary Material

Web_Material_uhag030

## Data Availability

The raw genome sequencing and RNA sequencing data from fluorescence, leaf, root, and shoot samples for genome annotation have been deposited in the China National Center for Bioinformation database under accession number PRJCA038347. The raw RNA sequencing data from HEO1 mutant and WT plants have also been deposited in the China National Center for Bioinformation database under accession number PRJCA047213. Files corresponding to the genome assembly, structural annotations, predicted protein sequences, and transcript sequences have been publicly deposited in Figshare (https://doi.org/10.6084/m9.figshare.30354568). The data supporting the findings of this work are available from the supplementary material or upon request.
