# Supplementary material for: Chromosome-level genome assembly of Origanum vulgare subsp. hirtum reveals evolutionary insights and regulatory modules in terpenoid biosynthesis
Source: Hortic Res. 2026 Jan 30;13(5):uhag030. doi: 10.1093/hr/uhag030 (PMC13161556; doi:10.1093/hr/uhag030)
Supplement: Web_Material_uhag030 [file web_material_uhag030.zip › S-figures for Ov20251230.pdf]

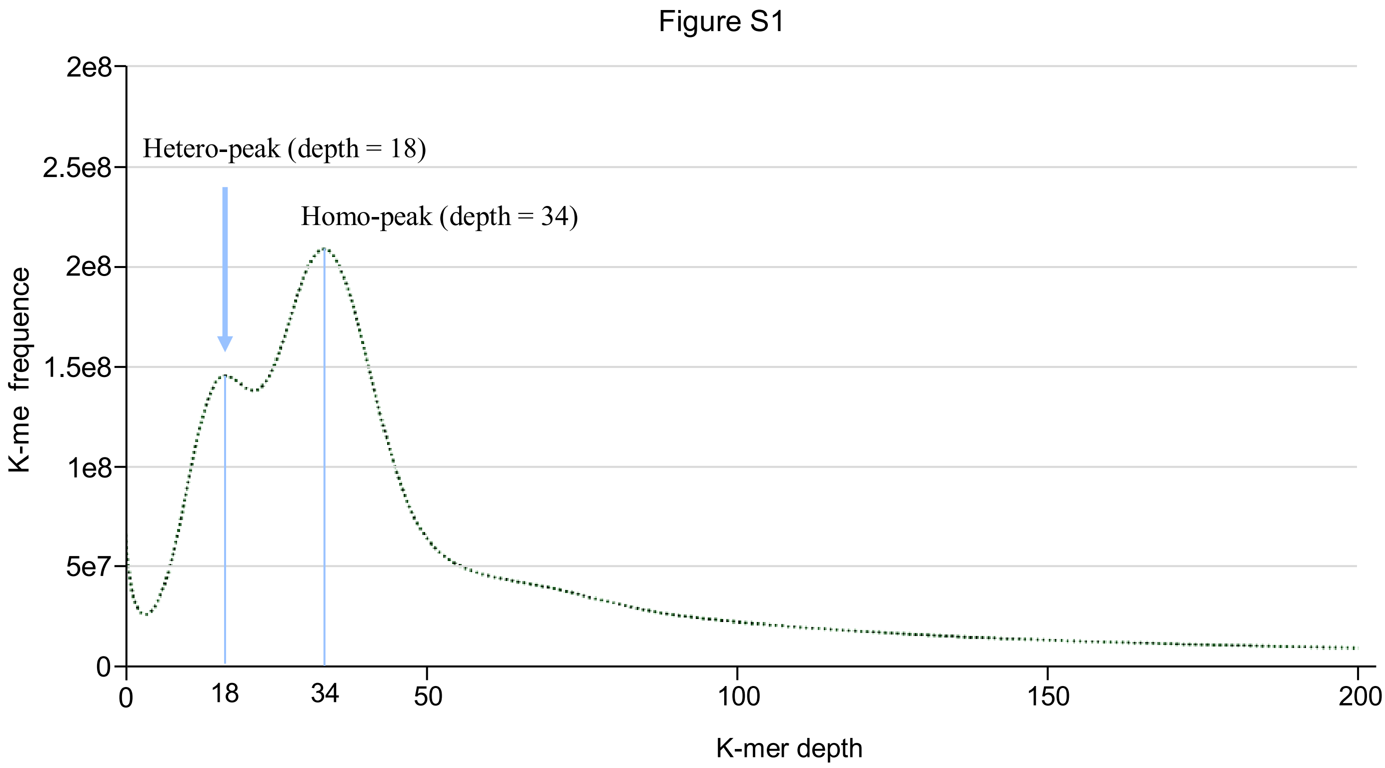


Figure S1. Diagram of K-mer frequency and depth.

Figure S2

a

| | Number (bp) | % of genome |
|---|---|---|
| A | 212,151,970 | 29.89 |
| T | 212,115,595 | 29.89 |
| C | 142,714,463 | 20.11 |
| G | 142,752,866 | 20.11 |
| N | 0 | 0.00 |
| total | 709,734,894 | -- |
| GC | 285,467,329 | 40.22 |

b

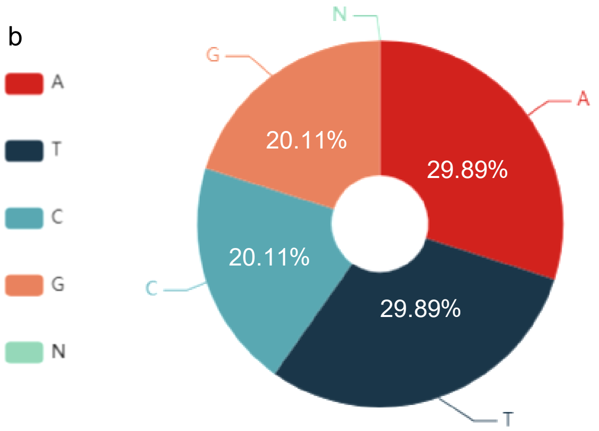


**Figure S2**. Number and percentage of four nucleotide bases (A, T, G, C) of the PacBio HiFi sequence data. The proportions of four nucleotide bases (A, T, G, C) are within normal ranges, with a combined GC content of 40.22% with no other bases (N) detected (0.00%).

Figure S3

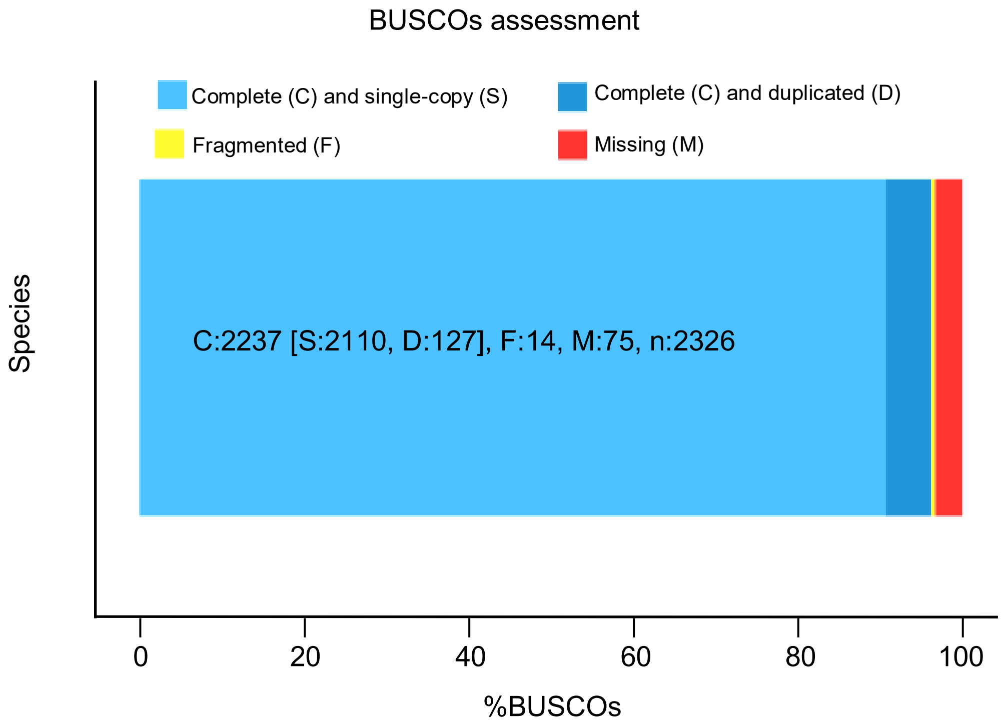


**Figure S3**. BUSCOs assessment to evaluate the assembly completeness of target genome. C: Complete BUSCOs; S: Complete and single-copy BUSCOs; D: Complete Duplicated BUSCOs; F: Fragmented BUSCOs; M: Missing BUSCOs; n: Total BUSCO groups searched.

Figure S4

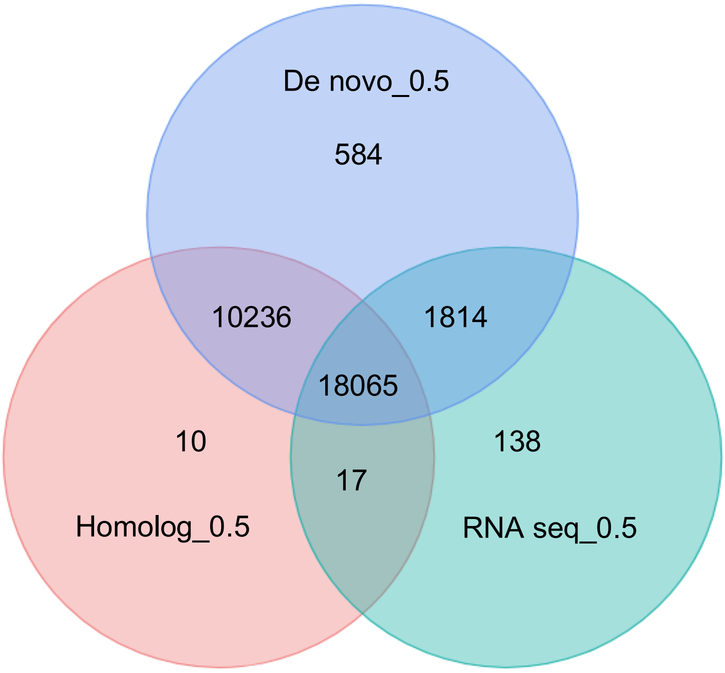


**Figure S4**. Venn diagram of identified genes through *De novo*, Homolog-based and RNA-seq.

Figure S5

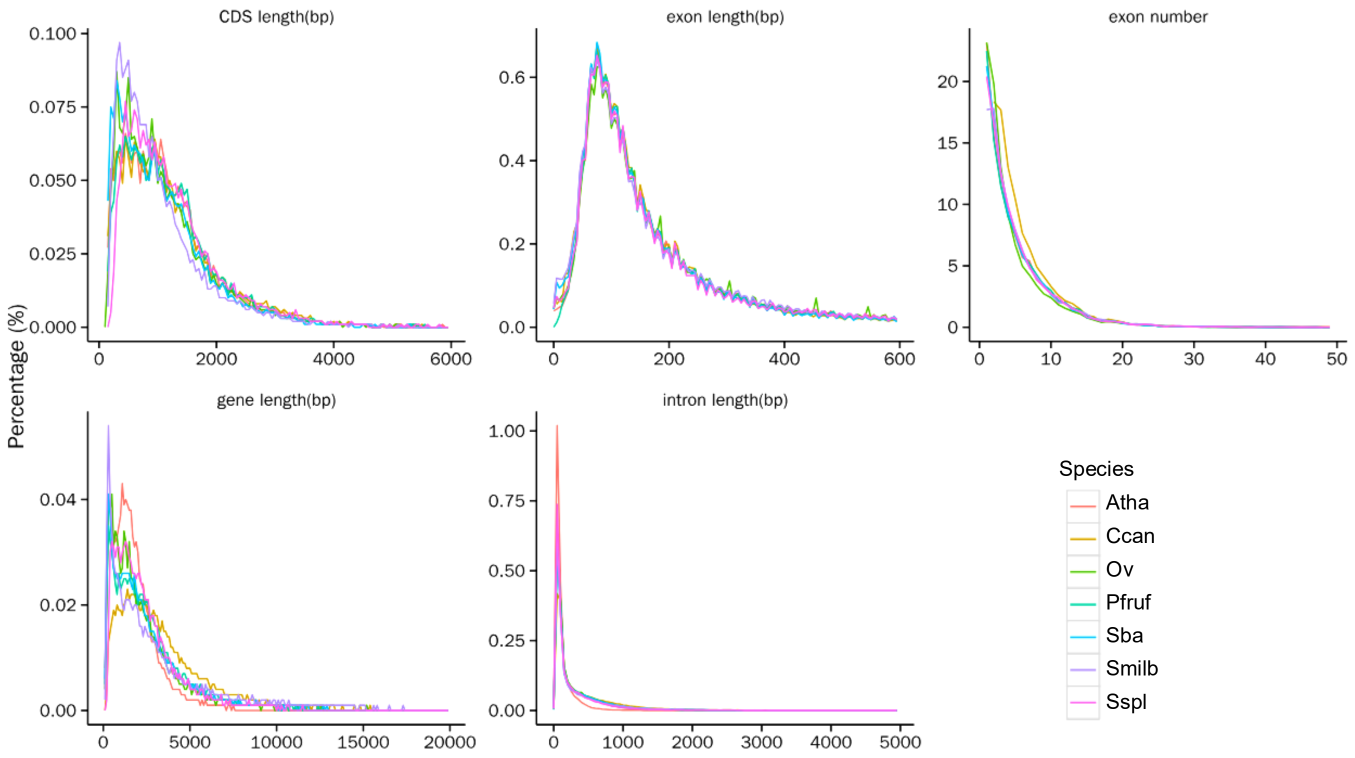


**Figure S5**. Gene features analysis in genomes of *O. vulgare* subsp. *hirtum* and its closely related species. Ov, *O. vulgare* subsp. *hirtum*; Sspl, *Salvia splendens*; Smilb, *Salvia miltiorrhiza*; Pfruf, *Perilla frutescens* var. *frutescens*; Sba, *Scutellaria baicalensis*; Ccan, *Coffea canephora*; Atha, *Arabidopsis thaliana*.

Figure S6

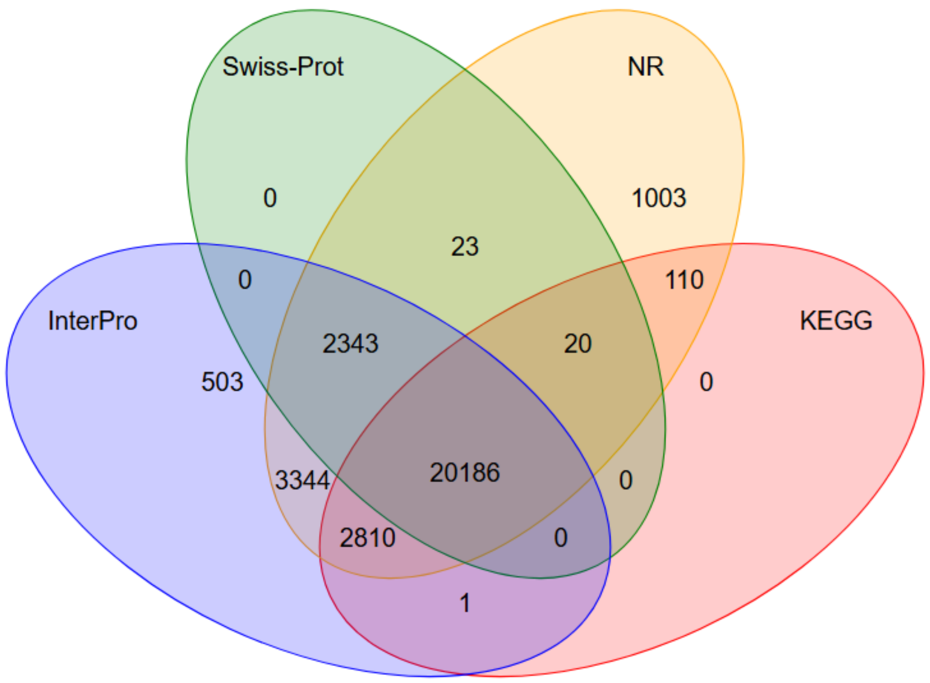


**Figure S6**. Functional annotation of genes in the genome of *O. vulgare* subsp. *hirtum* based on Swiss-Prot and nonredundant protein sequence database (NR), Kyoto encyclopedia of genes and genomes (KEGG), and integrative protein signature database (InterPro).

Figure S7

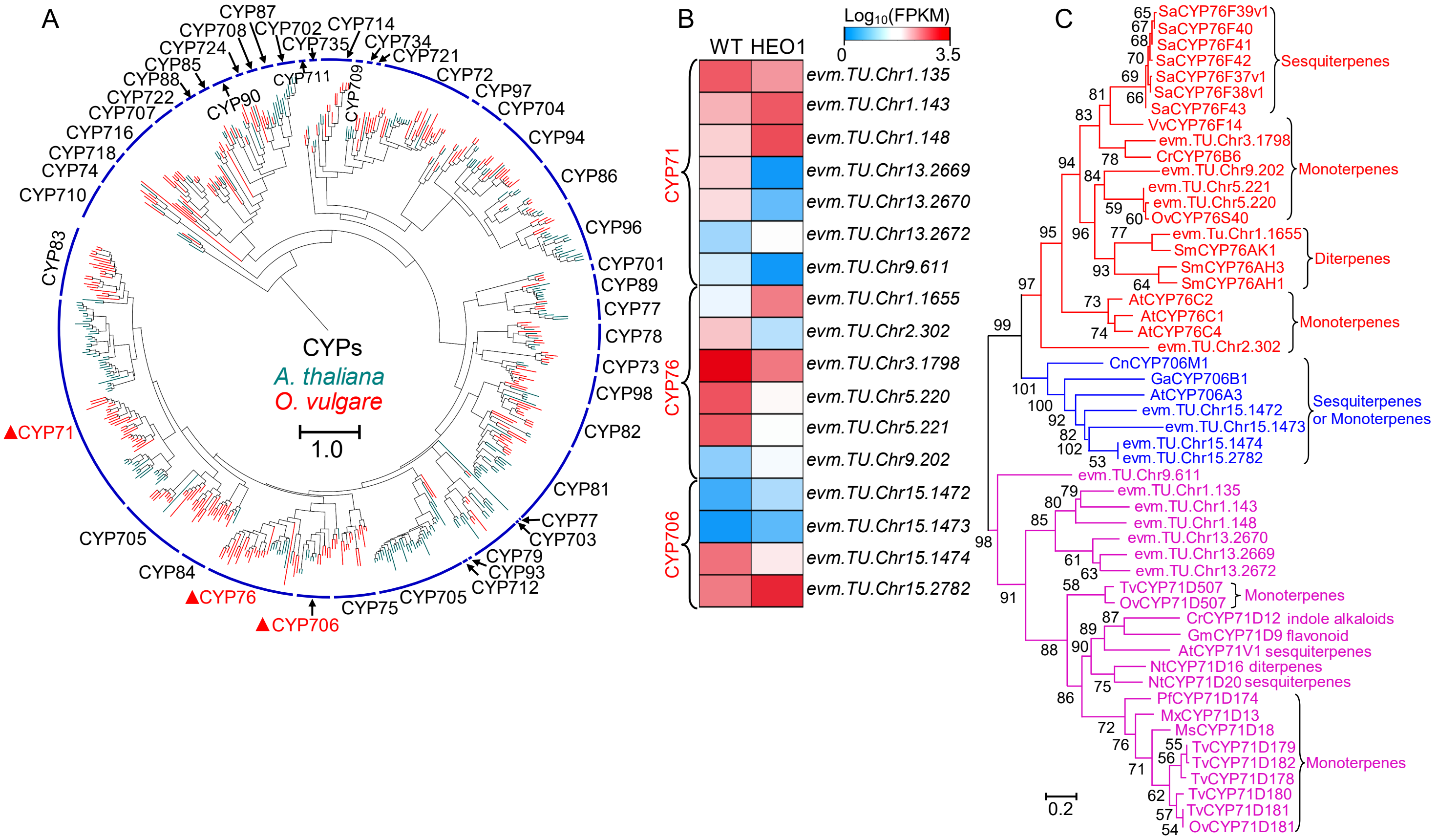

**Figure S7**. *In silico* predication of potential *CYP* genes involved in EO biosynthesis. (A) Phylogeny of CYPs identified in *O. vulgare* subsp. *hirtum* and *Arabidopsis* genomes. (B) Expression profiles of the differentially expressed oregano *CYP*s in the *CYP71*, *CYP76*, and *CYP706* subfamilies. (C) Evolutionary analysis of the differentially expressed *CYP*s using the Maximum Likelihood method with the highest log likelihood of -33235.61. This tree depicts the known and potential CYPs involved in thymol, carvacrol and thymohydroquinone biosynthesis in oregano (*O. vulgare*, Ov), *T. vulgaris* (Tv), *Arabidopsis thaliana* (At), *Callitropsis notkatensis* (Cn), *Catharanthus roseus* (Cr), *Gossypium arboretum* (Ga), *Glycine max* (Gm), *Mentha spicata* (Ms), *Mentha x piperita* (Mxp), *Nicotiana tabacum* (Nt), *Perilla frutescens* (Pf), *Santalum album* (Sa), *Salvia miltiorrhiza* (Sm), *Vitis vinifera* (Vv) (Krause et al., 2021). The scale bar indicates 0.2 amino acid substitutions *per* site.

Figure S8

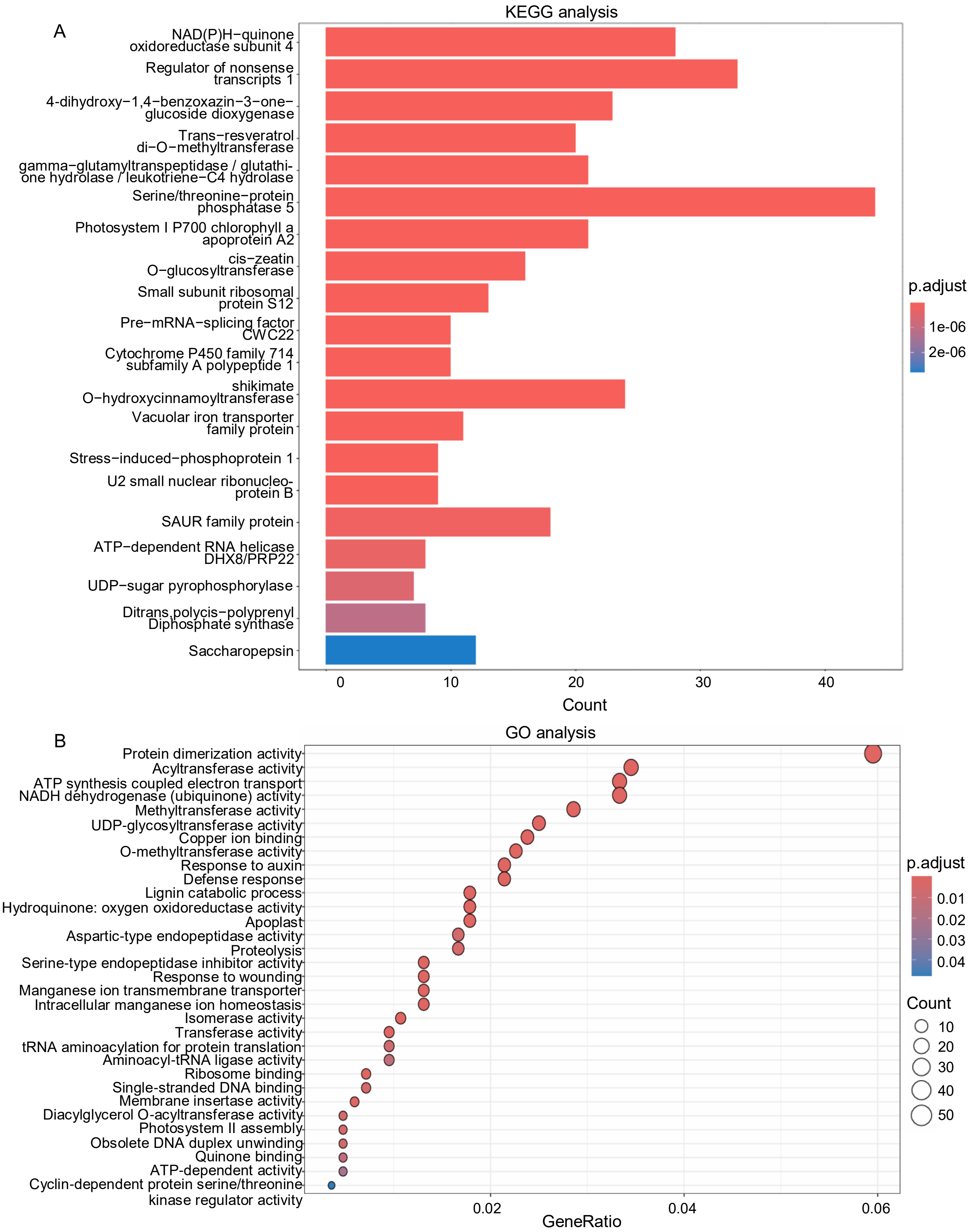


**Figure S8**. KEGG (a) and GO (b) enrichment analysis of expanded genes in Greek oregano.

Figure S9

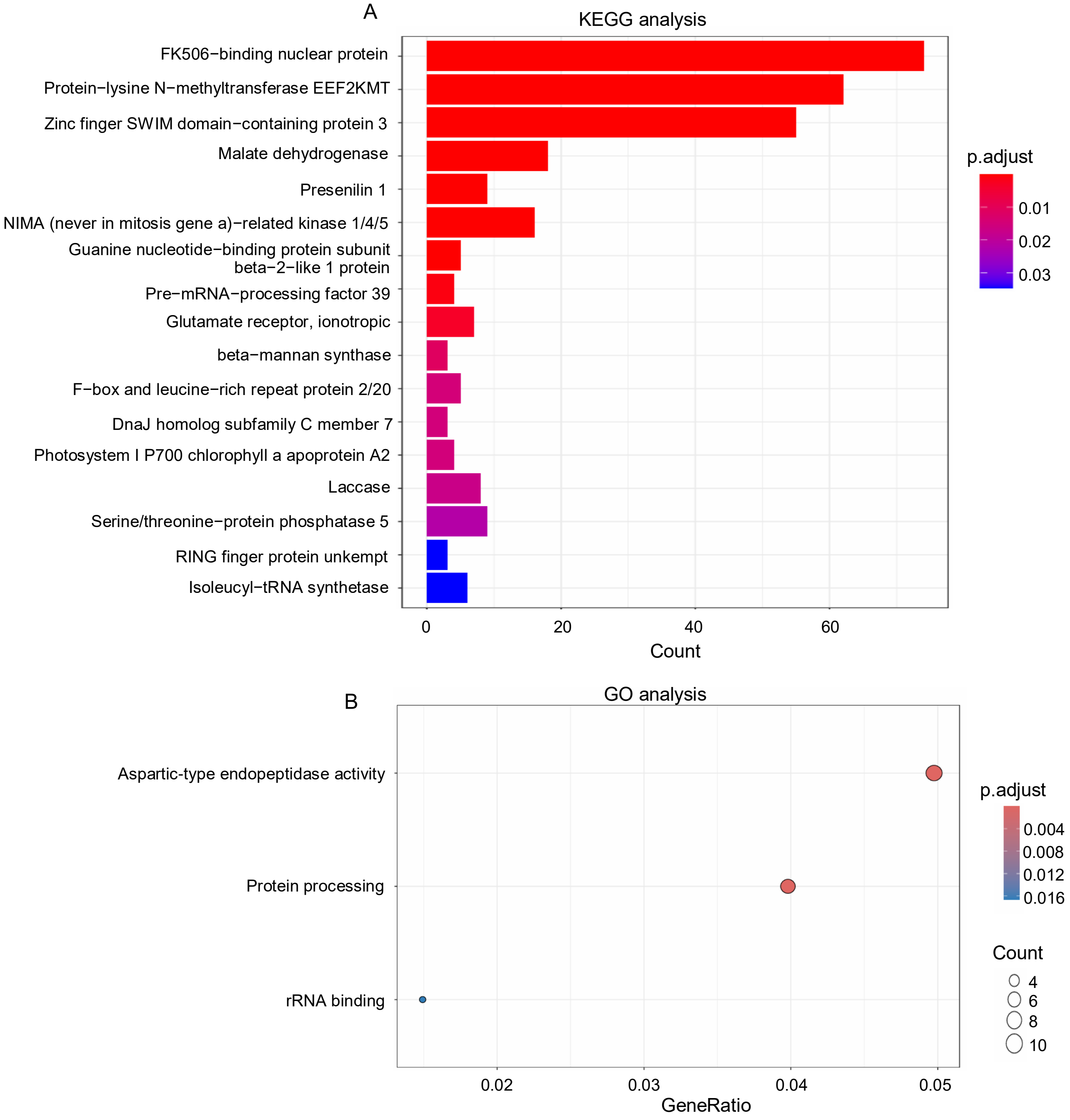


**Figure S9**. KEGG (a) and GO (b) enrichment analysis of specific genes in Greek oregano.

Figure S10

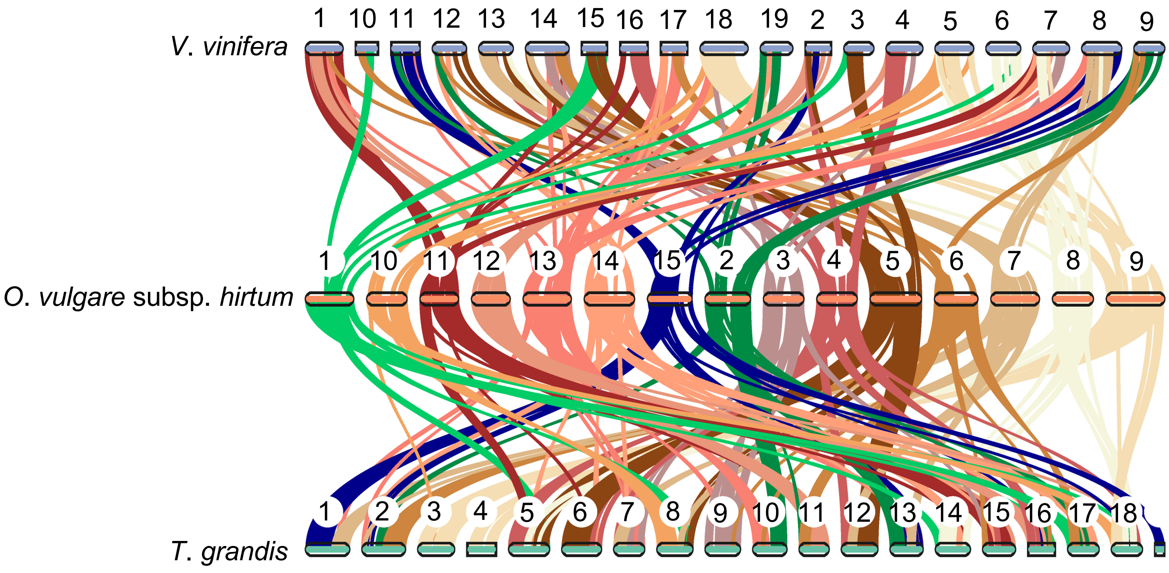


**Figure S10**. Chromosomal collinearity analysis among *O. vulgare* subsp. *hirtum*, *T. grandis*, and *V. vinifera*.

# Figure S11

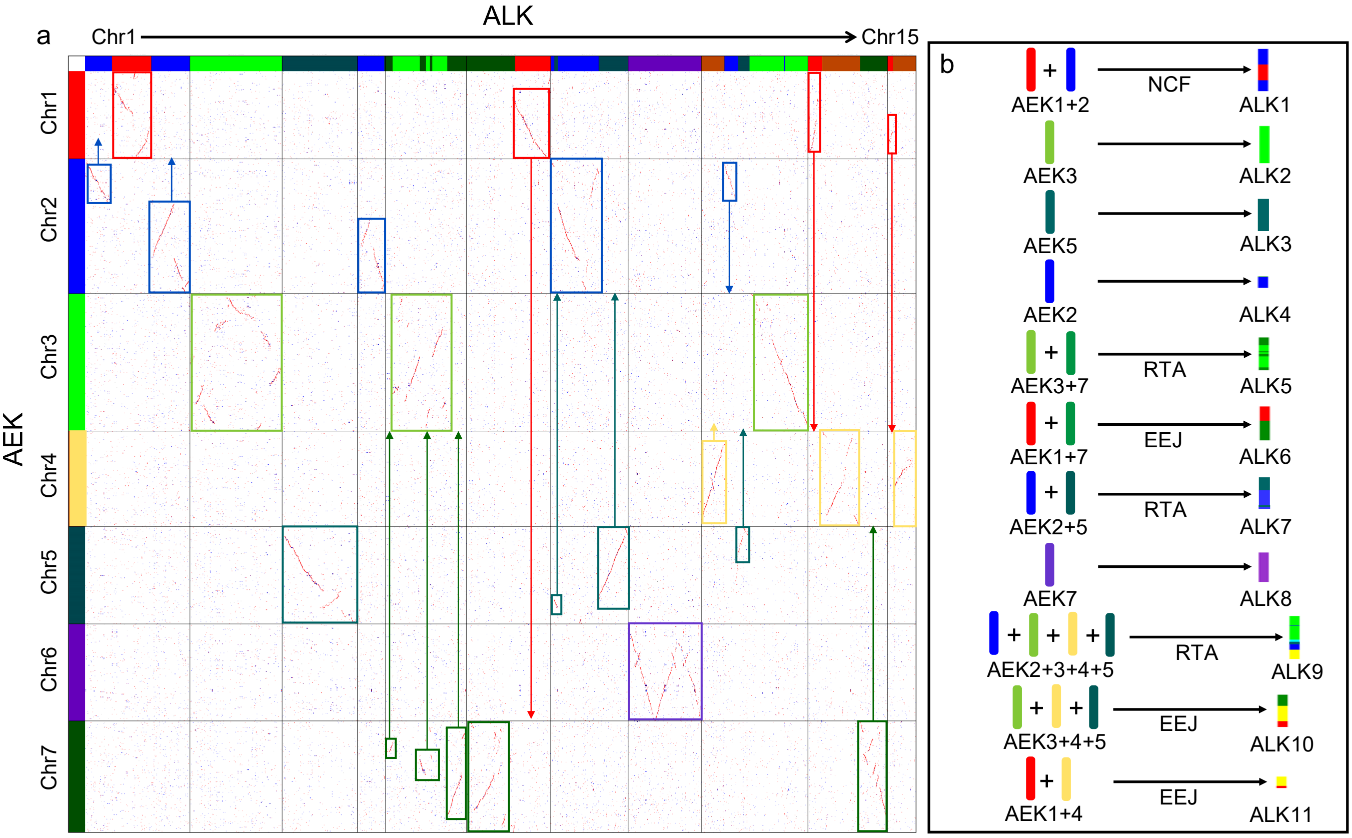


**Figure S11**. Karyotype evolutionary trajectory of ancestral eudicot karyotype (AEK) and ancestral Lamiales karyotype (ALK).

Figure S12

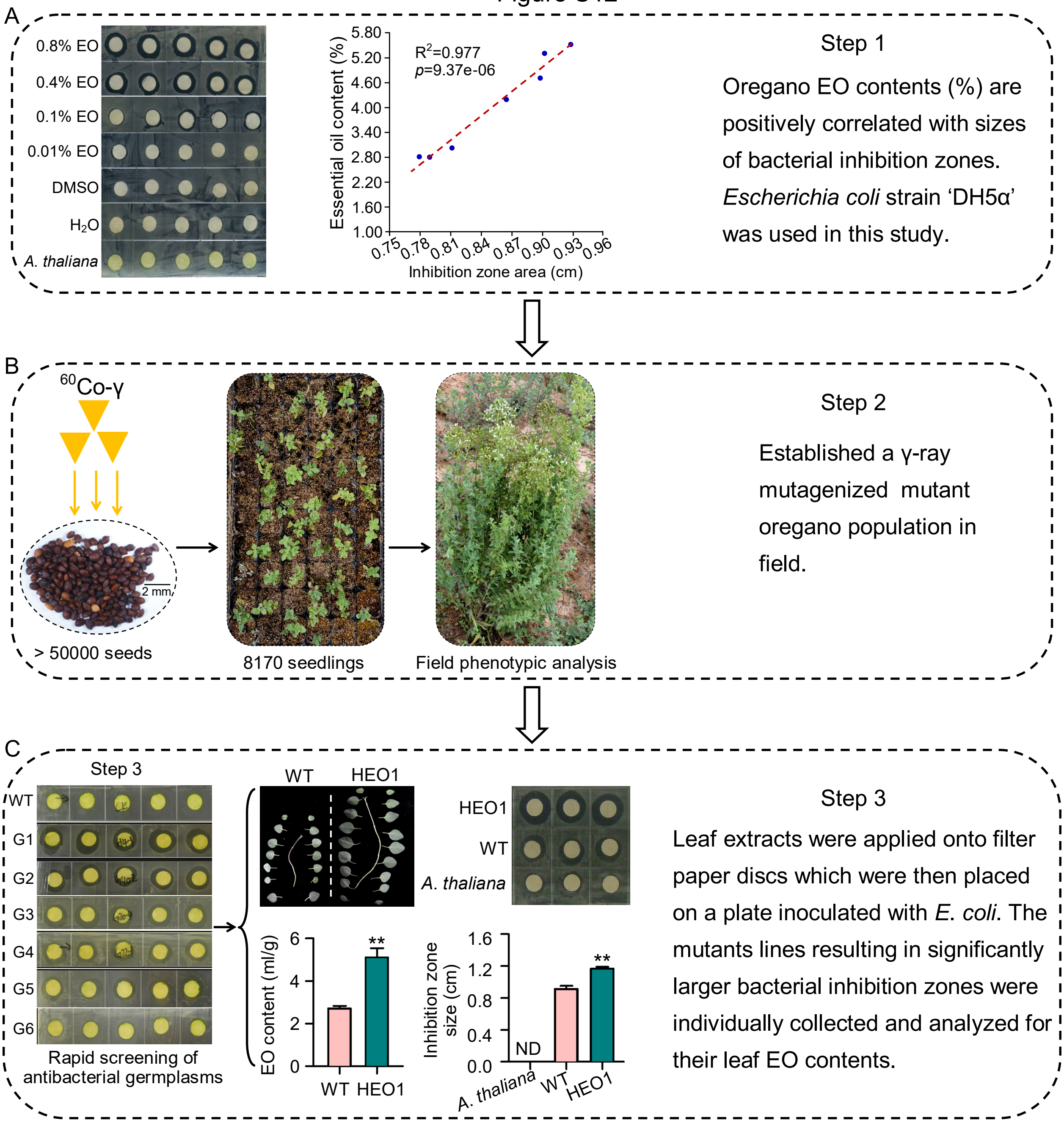


**Figure S12**. Illustration for procedures of Greek oregano mutant screening for high EO content. (A) Oregano EO has strong anti-bacterial effects and its EO contents (%) are positively correlated with sizes of bacterial inhibition zones.*Escherichia coli* strain 'T1' was used in this study. (B) A γ-ray mutagenized mutant population was established in the field. (C) From each mutant line, a defined amount of fresh leaf discs was ground. Then their leaf extracts were applied onto filter paper discs, which were then placed on a plate inoculated with *E. coli*. The mutant lines resulting in significantly larger bacterial inhibition zones were individually collected and analyzed for their leaf EO contents.

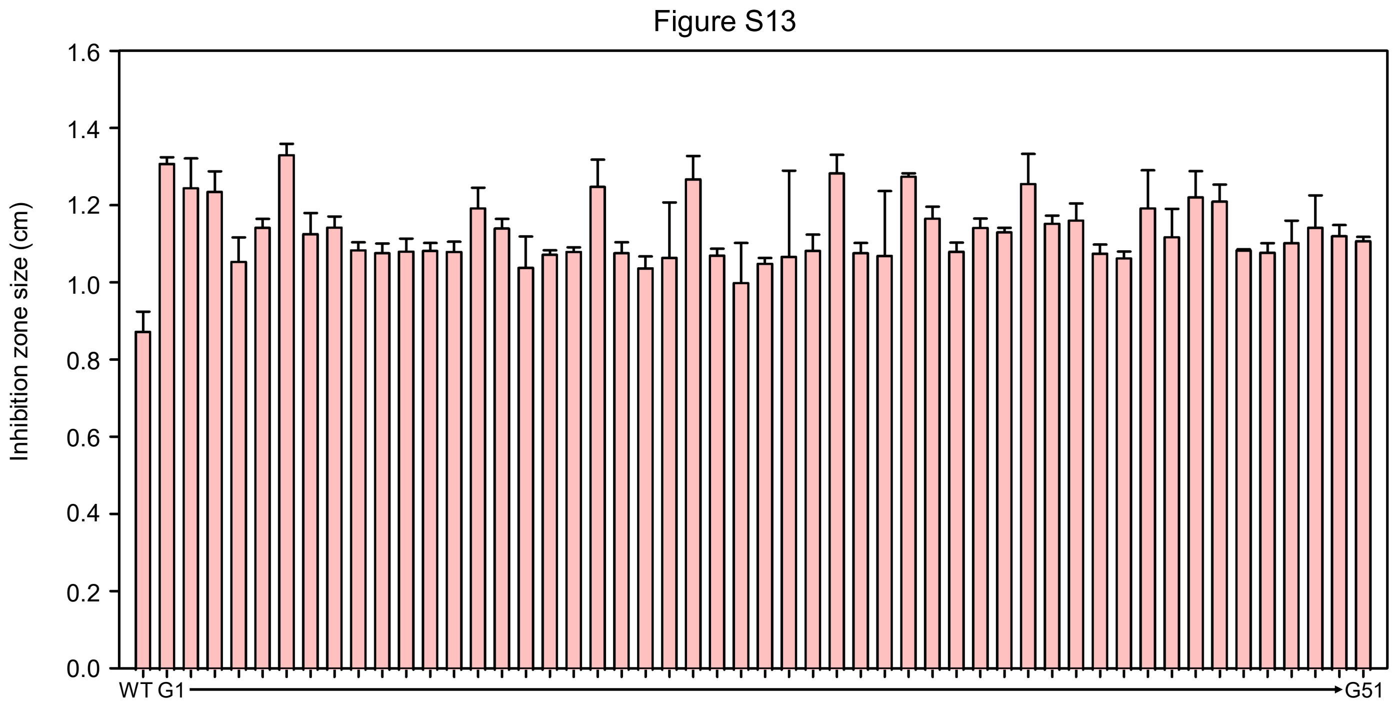


**Figure S13**. A total of 51 Greek oregano HEO mutants (G1 to G51) were identified with significantly larger sizes of bacterial inhibition zone than WT plant.

Figure S14

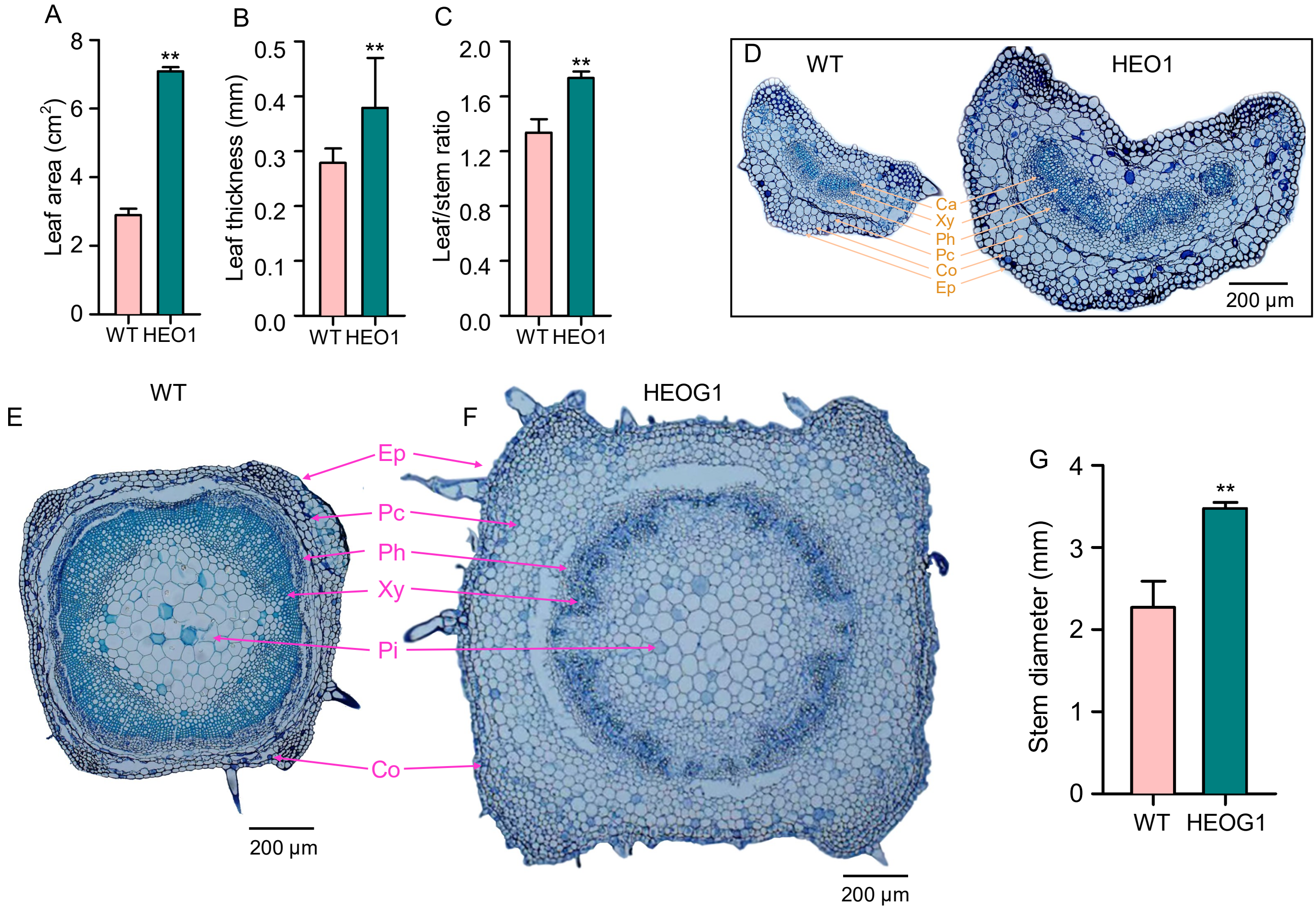


**Figure S14**. Morphology comparison between the HEO1 and the WT oregano. (A-C&G) Quantitative analysis of leaf area, leaf thickness, leaf-to-stem ratio, and stem diameter in HEO1 and WT. (D-F) Paraffin section image of petioles and stems of HEO1 and WT oregano. Note: Pc, parenchyma cells; Pi, pith; Ep, epidermis; Xy, xylem; Ph, phloem; Co, collenchyma; Ca, cambium; Pi: Pith part.

Figure S15

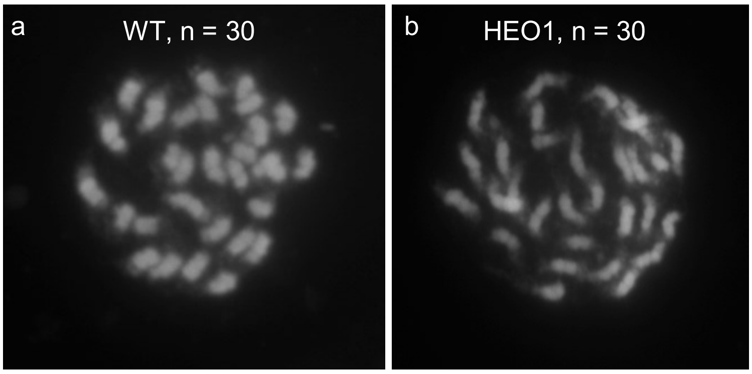


**Figure S15**. Chromosome numbers of the HEO1 and WT oregano.

Figure S16

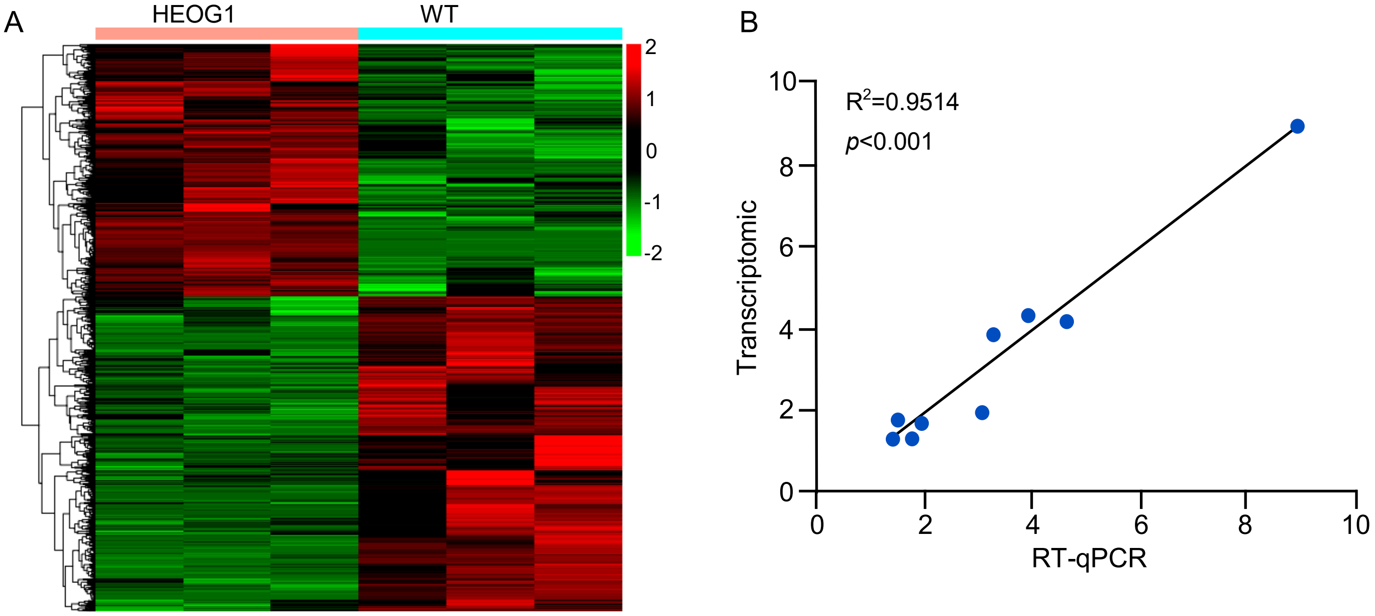


**Figure S16**. Heatmap diagram of differentially expressed genes between the HEO1 and WT oregano (A), and qRT-PCR verification of the transcriptomic data (B).
